# Transformation of flg22 perception into electrical signals decoded in vasculature leads to sieve tube blockage and pathogen resistance

**DOI:** 10.1126/sciadv.ads6417

**Published:** 2025-02-26

**Authors:** Alexandra C. U. Furch, Matthias R. Zimmermann, Gundula A. Noll, Lisa S. Wrobel, Sandra S. Scholz, Stefanie V. Buxa-Kleeberg, Jens B. Hafke, Judith Fliegmann, Axel Mithöfer, Katrin Ehlers, Tom Haufschild, Jonas Nötzold, Aline M. Koch, Veit Grabe, Florian Teutemacher, Jan-Peter Maaß, Dirk Prüfer, Ralf Oelmüller, Edgar Peiter, Karl-Heinz Kogel, Aart J. E. van Bel

**Affiliations:** ^1^ Department of Plant Physiology, Matthias-Schleiden-Institute for Genetics, Bioinformatics and Molecular Botany, Faculty of Biological Science, Friedrich-Schiller-University Jena, Dornburger Straße 159, D-07743 Jena, Germany.; ^2^ Institute of Plant Biology and Biotechnology, University of Münster, Schlossplatz 8, D-48143 Münster, Germany.; ^3^ Fraunhofer Institute for Molecular Biology and Applied Ecology IME, Schlossplatz 8, 48143 Münster, Germany.; ^4^ Institute of Phytopathology, Centre for BioSystems, Land Use and Nutrition, Justus Liebig University Giessen, Heinrich-Buff-Ring 26-32, D-35392 Giessen, Germany.; ^5^ Institute of Botany, Justus Liebig University Giessen, Heinrich-Buff-Ring 38, D-35392 Giessen, Germany.; ^6^ European School RheinMain, Theodor-Heuss-Straße 65, 61118 Bad Vilbel, Germany.; ^7^ Centre for Plant Molecular Biology, Eberhard-Karls-University Tübingen, D-72076 Tübingen, Germany.; ^8^ Research Group Plant Defense Physiology, Max-Planck-Institute for Chemical Ecology, Hans-Knöll-Straße 8, D-07745 Jena, Germany.; ^9^ Department of Cellbiology, Institute for Plant Sciences, University of Regensburg, Universitätsstraße 31, D-93053 Regensburg, Germany.; ^10^ Microscopic Imaging Service Group, Max-Planck-Institute for Chemical Ecology, Hans-Knöll-Straße 8, D-07745 Jena, Germany.; ^11^ Plant Nutrition Laboratory, Institute of Agricultural and Nutritional Sciences, Faculty of Natural Sciences III, Martin Luther University of Halle-Wittenberg, Halle (Saale), Germany.; ^12^ Institut de biologie moléculaire des plantes, CNRS, Université de Strasbourg, 12 rue du Général Zimmer, 67084 Strasbourg, France.

## Abstract

This study focuses on the question how and where information acquired by FLS2 perception of flg22 is transformed into electrical signals crucial for generation of local and systemic defense responses. In *Arabidopsis thaliana* and *Vicia faba* leaves, FLS2 density was high in the epidermis and vascular parenchyma, low in mesophyll, and absent in sieve elements (SEs). Aequorin-based examinations disclosed dual cytosolic Ca^2+^ peaks shortly after flg22 application, which corresponded with two voltage shifts from the epidermis to SEs. These signals were converted into rapid long-range action potentials (APs) or slower short-range variation potentials (VPs). Modified phytohormone-levels demonstrated systemic AP effects. Jasmonic acid up-regulation was significantly higher in wild-type than *Atseor1/2* mutants. Abundant Ca^2+^ influx associated with VPs was responsible for transient sieve element occlusion (SEO) near the flg22 perception site, whereas SEO was absent in *Atseor1/2* and *Atfls2* mutants. Biological relevance of SEO was demonstrated by higher susceptibility of *Atseor1/2* mutants to *Pseudomonas syringae* than wild-type plants.

## Teaser

Flg22-triggered dual electric signals are crucial for generation of local and systemic defense responses.

## INTRODUCTION

Adaptive plant defense requires recognition of conserved microbial molecules—so-called microbe-associated molecular patterns (MAMPs) (*1*)—or plant-derived molecules—so-called damage-associated molecular patterns (*2*), which induce appropriate defense responses. Early MAMP-induced intracellular signaling responses comprise modified transmembrane ion fluxes, an increase in cytosolic free calcium concentration ([Ca^2+^]_cyt_), membrane depolarization, activation of protein kinases, and the production of reactive oxygen species (ROS) (*3*).

In addition to local responses, perception of MAMPs leads to systemic spread of signals via the phloem [e.g., (*4*)]. Numerous systemic signals, such as diverse RNA species (*5*) and small proteins (*6*), nitric oxide and other ROS (*7*), azelaic acid (*8*), SFD1/GLY1-derived glycerol-3-phosphate (*9*), and dehydroabietinal (*10*), have been proposed to play integral roles in activating systemic defense responses in plants. Phytohormones {e.g., salicylic acid (SA) or jasmonates [jasmonic acid (JA) and JA-isoleucine conjugate (JA-Ile)]} also participate in systemic activation of defense responses via the phloem (*11*, *12*).

The flagellin epitope flg22 is a MAMP associated with bacterial infection. It is perceived by the receptor kinase FLAGELLIN SENSING 2 (FLS2) (*13*). The FLS2 receptor is active in diverse tissues and cell types, and its expression is induced by biotic and abiotic stresses, such as bacterial attack and wounding, and by the phytohormone SA (*14*). FLS2 receptors were frequently found not only in cell types exposed to bacterial entry, as in stomata or hydathodes, but also in the vascular system (*14*). Typical local responses of epidermal cells observed in flg22-exposed plants are the release of ROS (*15*), elevation of [Ca^2+^]_cyt_ (*14*, *16*), deposition of callose (*13*, *17*), and accumulation of defense proteins (*18*). Local flg22 application has also remote effects. Beck and co-workers (*14*) observed an increase in [Ca^2+^]_cyt_ in the vascular system of *Arabidopsis thaliana* seedlings after flg22 treatment, but the responding cell types were not identified with certainty. How the local perception of flg22 is transformed into a systemic signal is unclear, as are the location, nature, and propagation pathways of this signal.

The principal question raised here is whether flg22 perception also induces electropotential waves (EPWs) acting as systemic signals. EPWs have been studied mainly in response to abiotic stimuli (*19*, *20*), whereas less is known about their involvement in biotic stress responses (*21*, *22*). It seems that local Ca^2+^ signals, evoked by abiotic stimuli, can be transformed into phloem-located systemic EPWs associated with a transient halt of mass flow due to sieve element occlusion (SEO) in *Vicia faba* (*19*, *21*). The transient occlusion consists of an immediate protein plugging of sieve pores, followed by a slower sieve pore constriction through callose deposition (*19*, *23*, *24*). The question now arises whether and in which way phytopathogen perception lastly results in EPW propagation associated with temporary SEO and whether and how EPWs are interconnected with chemical systemic signaling.

The first issue addressed was the exact cellular location(s) and densities of FLS2 sensors in *A. thaliana* midribs to complement a previous study (*14*). Using *VfFLS2* gene expression results and sieve element protoplasts, FLS2 localization was also assessed in *V. faba* as a more amenable and optically more conclusive alternative to *A. thaliana*. As the next step, local Ca^2+^ patterns in flg22-challenged cells and the associated electrical responses were recorded in various ways. The deployment patterns of FLS2 receptors, flg22-induced voltage shifts, and plasmodesmal connectivity offer the potential for electrical signaling from the epidermis to sieve elements (SEs). Passage of electropotential disbalances brought about by flg22 application was measured by extracellular and intracellular recordings in this area of leaf veins of *A. thaliana* and *V. faba*. Furthermore, resultant EPWs in SEs were recorded and identified. EPWs may cause tidal Ca^2+^ waves through SEs, triggering transient sieve plate plugging and/or occlusion (*19*, *25*) and modified phytohormone synthesis (*26*, *27*). Therefore, systemic effects of flg22 application on SEO and on phytohormone synthesis were investigated.

## RESULTS

### FLS2 receptor density varies between *A. thaliana* midrib cell types

**Fig. 1.**
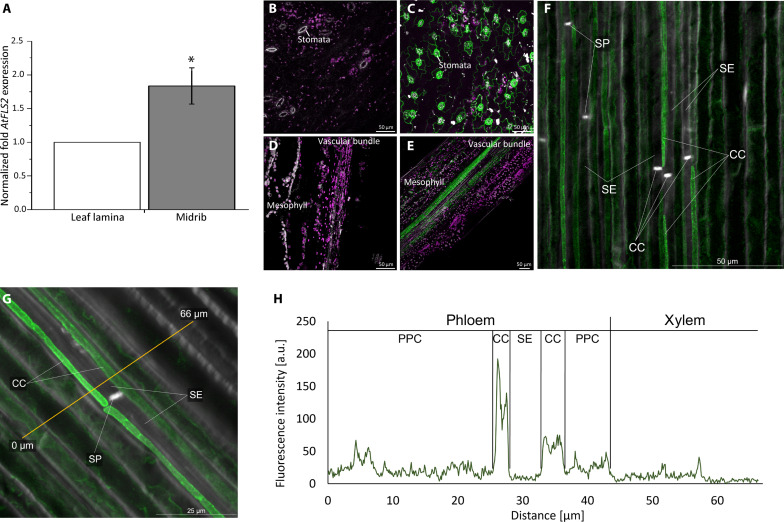
Cellular deployment of FLS2 receptors in *A. thaliana* leaf tissues. (**A**) Relative expression levels of FLS2 in midrib tissue were significantly (*P* < 0.05) higher than in the rest of the lamina. The values were normalized to the housekeeping gene (actin), and the whole leaf sample and were defined as 1.0 (*n* = 4). (**B** to **G**) For determination of the intercellular location of FLS2-receptors, pFLS2::FLS2-3xmyc-GFP plants [(C), (E), (F), and (G)] were examined and analyzed by CLSM. Pictures of WT Columbia [(B) and (D)] of leaf epidermis surface (B) and longitudinal minor vein sections (D) were used as controls. In the pFLS2::FLS2-3xmyc-GFP, the epidermis of the abaxial leaf side shows a strong GFP fluorescence at the stomata (C). [(D) to (G)] Longitudinal sections of the midrib and [(F) and (G)] a detail of the vascular bundle including companion cell (CC), sieve element (SE), sieve plate (SP), and phloem parenchyma cells (PPC). In CCs and PPCs, the GFP fluorescence (green) is very strong, whereas cortex cells, the midrib epidermal cells, and notably SEs show less GFP fluorescence. In the detailed pictures of the vascular tissue [(F) and (G)], the localizing signal was enhanced by the use of a GFP antibody in combination with Alexa Fluor 488 secondary antibody. The magenta color represents autofluorescence of chloroplasts, and the gray color represents the thick cell wall material [in (F) and (G) stained with Fluorescent Brightener]. The experiment was repeated with five plants. (**H**) Quantification of the GFP fluorescence along a line (yellow) of interest (G). a.u., arbitrary units.

A previous FLS2 receptor localization study (*14*) focused on the plant and tissue level, but neither revealed the cellular identity nor the quantity of receptors in different cell types. We determined the *AtFLS2* gene expression level in the leaf lamina and midrib tissue by reverse transcription quantitative polymerase chain reaction (RT-qPCR) (Fig. 1A; *n* = 4) and examined the distribution of FLS2 receptors between midrib cells in an *A. thaliana* line harboring a green fluorescent protein (GFP)–tagged FLS2 [*pFLS2::FLS2*-3xmyc-GFP; (*28*)] by confocal laser scanning microscopy (CLSM) (Fig. 1, B to G; *n* = 5).

We found significantly (*P* < 0.05) higher gene expression levels of *AtFLS2* in midrib tissue in comparison to lamina tissue of 5-week-old *A. thaliana* plants (Fig. 1A). Microscopic localization confirmed an appreciable FLS2-GFP density in guard cells and epidermal cells [Fig. 1, B and C; cf. (*14*)] and disclosed high FLS2-GFP abundance in the vascular area, especially in companion cells, and phloem and xylem parenchyma cells (Fig. 1, D to H). Hardly any FLS2-GFP was detected in SEs (Fig. 1, F to H).

### Flg22 triggers biphasic Ca^2+^ influx into *A. thaliana* midrib cells

**Fig. 2.**
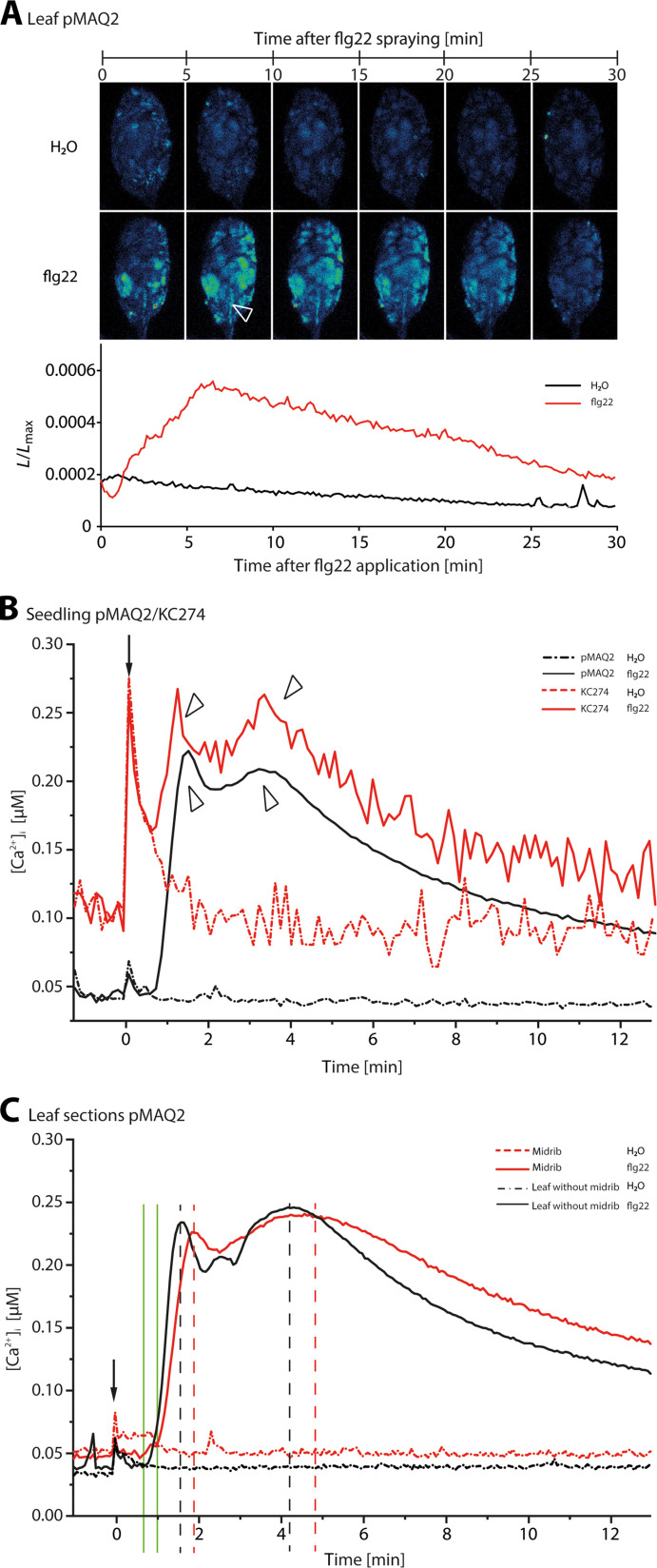
Flg22-induced changes of intracellular Ca^2+^ [Ca^2+^]_cyt_ in the entire seedlings, leaves, and vascular and laminar sections of *A. thaliana*. (**A**) An flg22 solution [(1 μM in double-distilled water (H_2_Odd)] or H_2_Odd (as a control) was sprayed onto the leaf surface of an intact *A. thaliana* plant expressing apoaequorin (pMAQ2). The [Ca^2+^]_cyt_ change was recorded as successive luminescence images of [Ca^2+^]_cyt_-dependent photon counts accumulated every 300 s. The [Ca^2+^]_cyt_-dependent photon counts are presented as *L*_max_-normalized values (luminescence counts per s/total luminescence counts remaining). The [Ca^2+^]_cyt_ increased during the first 5 to 6 min in delimited areas, scattered over the lamina and main vein (marked with a white arrow head; *n* = 3). (**B**) The specific [Ca^2+^]_cyt_ change in the vascular system was further investigated by comparing the response to flg22 application of the pMAQ2 line and the vasculature-limited GAL4 enhancer trap line KC274. The entire seedlings were treated with 1 μM flg22 or water, and the induced Ca^2+^-dependent luminescence was recorded using a luminometer. After flg22 treatment, both lines showed two successive Ca^2+^ peaks (marked with arrow heads): The first maximum occurred 1 min after flg22 application followed by a second one, 3 to 4 min later (*n* = 8). (**C**) To further discriminate the time-staggered response of the laminar and vascular tissues to flg22, midribs only and leaves from which midribs were excised were analyzed. The time shift of the dual Ca^2+^ responses in midribs and leaves without the midrib is marked with green vertical lines. Midribs only showed a delayed response as compared to leaves without midribs (peaks are marked with vertical dashed lines; *n* = 20), which suggests a retarded arrival of the Ca^2+^ influx response in vascular tissue. The application of flg22 or H_2_O is marked with an arrow. Both result in peak upon application, which is more pronounced in the KC274 line, likely due to the high sensitivity of the *Apoaequorin*-expressing vascular cells to mechanical stimulation.

MAMP binding to receptors is commonly followed by a transient elevation of cytosolic Ca^2+^ ([Ca^2+^]_cyt_), initiated by activation of Ca^2+^-permeable channels (*29*). Because flg22 application induced an instant Ca^2+^ influx (*14*), we assessed more in detail the effect of flg22 on the [Ca^2+^]_cyt_ level in the Ca^2+^ reporter line pMAQ2 (*30*) expressing aequorin in the cytosol of all cell types. Upon spraying of 1 μM flg22 onto pMAQ2 leaves, [Ca^2+^]_cyt_ increased during the first 5 min in scattered areas of the lamina and the midrib (Fig. 2A; *n* = 4). Such a patchiness has been observed previously upon exposure to other stimuli and upon aequorin discharge and is likely caused by heterogeneous aequorin reconstitution (*31*).

To discriminate [Ca^2+^]_cyt_ accumulation in vascular and mesophyll cells, flg22 was sprayed onto the vascular cell–specific GAL4 enhancer trap line KC274, which expresses *APOAEQUORIN* only in the vascular system (*32*). Consistent with the results obtained with pMAQ2, 1 μM flg22 induced a rapid [Ca^2+^]_cyt_ increment in KC274 seedlings (Fig. 2B; *n* = 8). The [Ca^2+^]_cyt_ level rose to a first peak within 30 to 60 s, followed by a second peak between 2 and 3 min after flg22 application (Fig. 2B). When the measurement interval was decreased to 4 s, a response delay of ~20 s between KC274 and pMAQ2 showed up (fig. S1), indicating a faster Ca^2+^ response of epidermal cells to flg22 in comparison to vascular cells. This slower response of vascular cells was also found by comparing *A. thaliana* leaves without the midrib and excised midribs (Fig. 2C; *n* = 20). The effect of flg22 application was expected to be quicker in leaves without the midrib than in midribs only, where the average recordings of the response will be slower given the very low density of FLS2 in midrib epidermal cells (Fig. 1E). This approach confirmed the biphasic Ca^2+^ influx and a delay of Ca^2+^ influx in midrib cells as compared to the rest of the leaf.

### Flg22 elicits electrical signaling from the epidermis to vasculature in *A. thaliana*

In intact leaves, stomata seem to be the principal flg22 recognition sites due to the high FLS2 abundance in guard cells and epidermal cells (Fig. 1C) (*14*). Consequently, the collective sensing capacity by FLS2 in the epidermis cells may meet that of the guard cells. Lack of FLS2 receptors in mesophyll and SEs and the delay of Ca^2+^ influx into vascular tissues (Fig. 2C) suggests the propagation of a signal, which may be of an electrical nature, from the epidermis to vascular tissues, enabling of crossing the FLS2-poor mesophyll area (Fig. 1). At the SE borderline, this lateral electrotonic wave may be transformed into an EPW along the SEs (*19*, *23*).

**Fig. 3.**
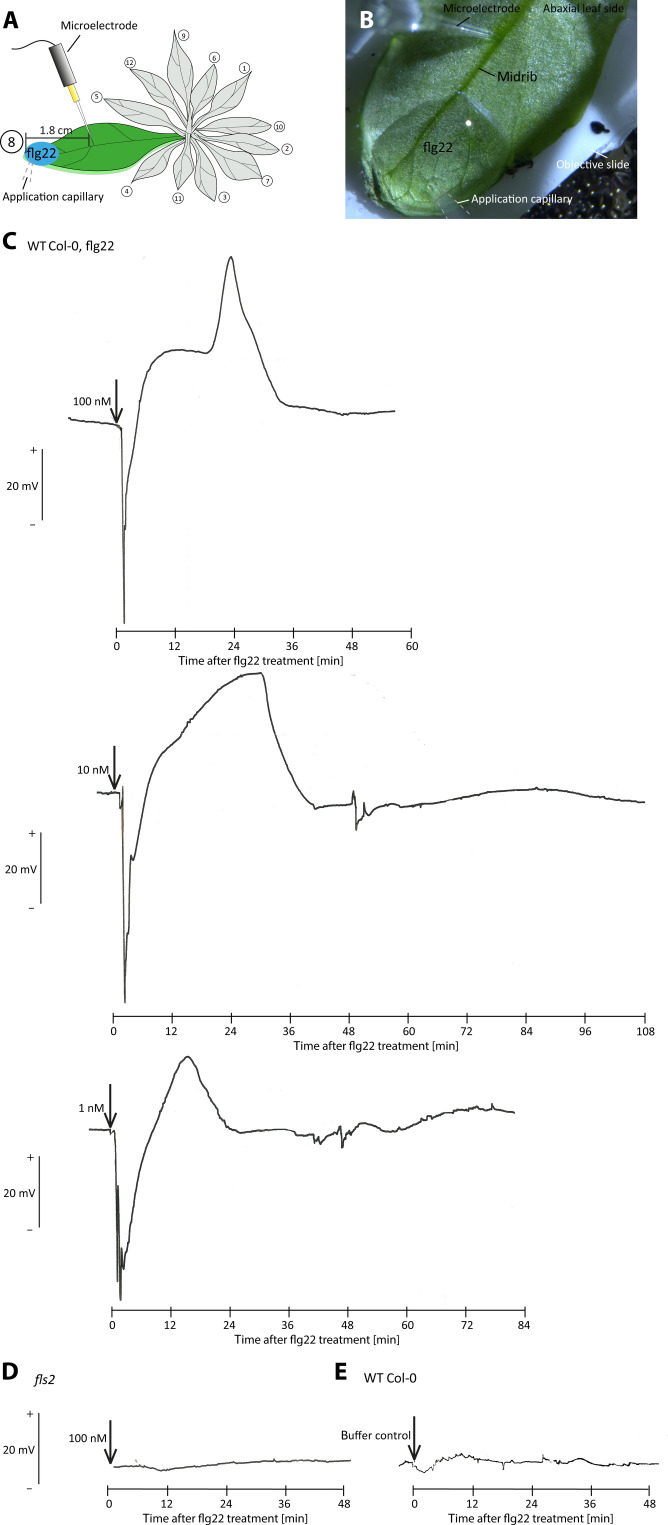
Extracellular voltage recordings in response to remote epidermal flg22 application onto the *A. thaliana* midrib. (**A** and **B**) The tip of a microelectrode was pierced blindly into the midrib of the abaxial leaf side and used to detect voltage shifts to remote application of 10 μl of flg22 (1, 10, or 100 nM administered separately to leaves of different plants) dissolved in a bathing medium containing 0.1% Tween 20. The flg22 solutions or a bathing medium (control) were carefully dropped onto the abaxial epidermis. (**C**) Flg22 induced voltage shifts in *A. thaliana* WT plants but not in (**D**) *fls2* mutant plants or (**E**) after a control treatment. Time points of flg22 application are marked with an arrow. Each measurement was repeated at least four times.

To test this hypothesis, voltage changes were recorded extracellularly in the leaf midrib in response to flg22 application at the intact leaf tip (Fig. 3, A and B). Ten to 30 s after application of flg22 (1, 10, and 100 nM) onto the lower leaf surface, electrical signals were measured at a distance of ~20 mm from the application site. Given the considerable distance to the site of flg22 application, these profiles likely represent EPWs. A strong (45 to 65 mV) but short (4 to 6 min) depolarization was immediately followed by a hyperpolarization (20 to 47 mV) lasting ~20 to 45 min (Fig. 3C; *n* = 4 to 6 biological replicates). The hyperpolarization consisted of two overlapping peaks, the second of which became increasingly dominant with the flg22 concentration (Fig. 3). The hyperpolarizations were not detected in an *fls2* knockout mutant (Fig. 3D; *n* = 5 biological replicates) or in response to application of a bathing medium (Fig. 3E; *n* = 7 biological replicates). An additional set of measurements is shown in fig. S2. In conclusion, flg22-induced voltage shift messages are not only able to overcome the FLS2-poor gap between the epidermis and vascular tissue but also able to propagate over longer distances after a transformation in EPWs.

### Flg22 induces transient blockage of phloem mass flow in *A. thaliana*

EPWs, associated with “tidal waves” of Ca^2+^ influx into SEs (*19*, *25*, *33*), trigger two mechanisms of blocking phloem mass flow in *V. faba* (*19*, *23*) and *Cucurbita* (*24*) viz. SEO by protein plugging and constriction by callose deposition around the sieve pores. These events appear to be universal among eudicotyledons (*21*) and can occur alone or in tandem depending on the stimuli, leading to Ca^2+^ influx [e.g., (*23*, *34*)].

**Fig. 4.**
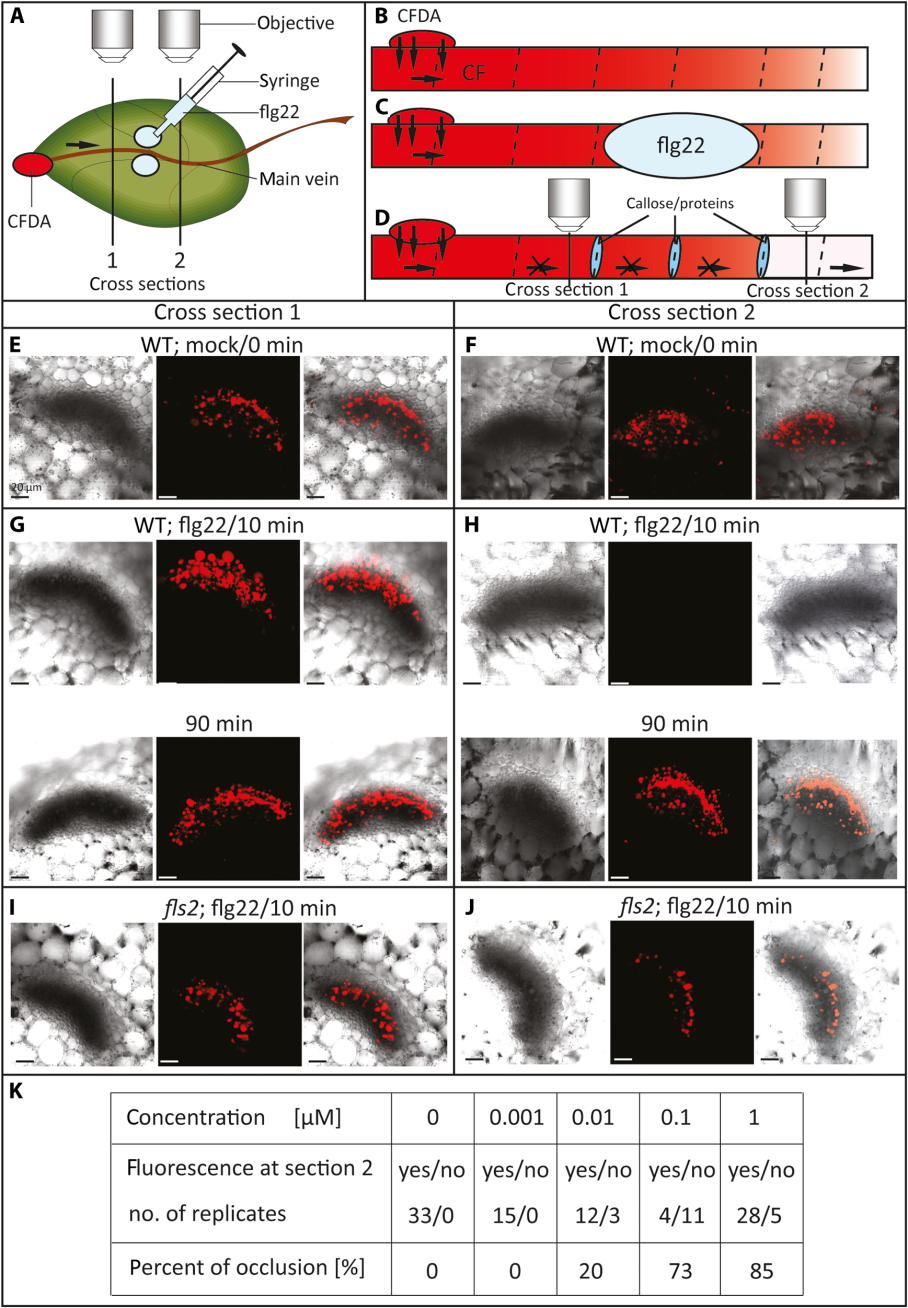
Examination of a transient stop of mass flow in sieve tubes in *A. thaliana* WT and *fls2* leaves after infiltration of flg22. (**A** to **D**) Schematic drawings of the experimental setup and SE reactions. (A) The nonfluorescent ester CFDA (membrane permeable) was continuously applied to cropped leaf tips and trapped in SEs. There, it was cleaved by esterases to form the polar (membrane impermeable) fluorescent CF. Transport of CF was observed by CLSM at cross sections (vertical lines) upstream (1) and downstream (2) the flg22 infiltration site. Control plants (mock) were treated with a bathing medium without flg22. (B) CF was transported by mass flow through sieve tubes. (C) After 2 hours, 100 μl of 1 μM flg22 was pressure infiltrated via a 1-ml syringe, 0.5 cm right and left of the midrib in leaf (D). At different time points after flg22 infiltration, CF fluorescence in the phloem was examined in cross sections upstream (**E**, **G**, and **I**) and downstream (**F**, **H**, and **J**) the infiltration site. SEO is marked by blue ovals (D). [(E) and (F)] In mock-treated plants, CF fluorescence was always detected at both sides, upstream and downstream the infiltration site (fig. S2). [(G) and (H)] Ten minutes after flg22 treatment, no CF fluorescence was observed downstream the flg22 infiltration site of WT plants, indicative of SEO. Ninety minutes after flg22 infiltration, CF fluorescence was detected again in both cross sections, which disclosed lifting of SE blockage and resumption of phloem transport. [(I) and (J)] In the *fls2* mutant, CF fluorescence was always detected upstream and downstream of the flg22 infiltration site, showing undisturbed mass flow due to compromised perception of flg22 (exemplarily shown for 10 min after flg22 infiltration; *n* = 5). Transmission channel, 488-nm line, and merged image are presented from left to right [(E) to (J)]. (**K**) Summary of SEO induced with different flg22 concentrations.

To test whether flg22-induced EPWs trigger SEO, we first applied continuously nonfluorescent 5(6)-carboxyfluorescein diacetate (CFDA) onto cropped leaf tips of wild-type (WT) and flg22-insensitive *fls2 A. thaliana* plants. Downstream transport of trapped fluorescent carboxyfluorescein (CF) through the sieve tubes was recorded by CLSM (Fig. 4). After ~2 hours, when the CF front had moved downward through the leaf, a drop of 100 μl of 1 nM to 1 μM flg22 was infiltrated through the epidermis flanking the midrib (Fig. 4, A to K).

In mock treatments of WT plants (bathing media without flg22), CF fluorescence in the sieve tubes was detected at either side of the application site (Fig. 4, E and F, and fig. S3; *n* = 8). By contrast, using 1 μM or 100 nM flg22, no fluorescence was observed downstream of the application site at 10 min after flg22 treatment of WT leaves, indicating that SEO instantly impaired mass flow across the flg22-treated region (Fig. 4, G, H, and K; *n* = 15 to 33). Ninety minutes after flg22 treatment, CF fluorescence was again detected in sieve tube cross sections, which demonstrated lifting of the barriers in sieve tubes. Application of 10 nM flg22 rarely induced a stop of mass flow, and 1 nM flg22 did not trigger an occlusion (Fig. 4K; *n* = 15).

In sheer contrast, flg22-induced SEO was not observed in the *fls2* mutant (Fig. 4, I and J; *n* = 25). Moreover, *Atgsl7* and *Atseor1/2* plants did not occlude sieve tubes after flg22 infiltration (fig. S4; *n* = 5), indicating a participation of callose and SEOR proteins in the blockage. Fluorescence was detected continuously upstream and downstream of the application site for several hours after flg22 treatment in both *A. thaliana* mutants.

### Identification and characterization of an FLS2 ortholog in *V. faba*

Apparently, flg22-triggered SEO is initiated by FLS2 receptors of *A. thaliana*. Can this phenomenon be observed even more discretely in *V. faba*? This species is more amenable to physical manipulations due to the large SE size, protocols for isolation of phloem cell types such as companion cells and SEs are available (*35*), intracellular voltage recordings with a precise reference to the cell type are feasible (*19*, *23*), EPW-triggered occlusion mechanisms can be assessed more clearly (*19*, *23*, *33*), and it harbors forisomes in SEs (*36*, *37*). Forisomes are giant spindle-shaped protein bodies in legume SEs, which disperse in response to sufficient Ca^2+^ influx and by doing so occlude the sieve pores and halt mass flow. Their behavior thus allows us to gain quantitative optical information on the effect of distant flg22 application on Ca^2+^ shifts in SEs.

The major disadvantage of *V. faba* over *A. thaliana*, however, is the lack of mutants. The existence of VfFLS2 residing in the epidermal plasma membrane has not yet been demonstrated.

**Fig. 5.**
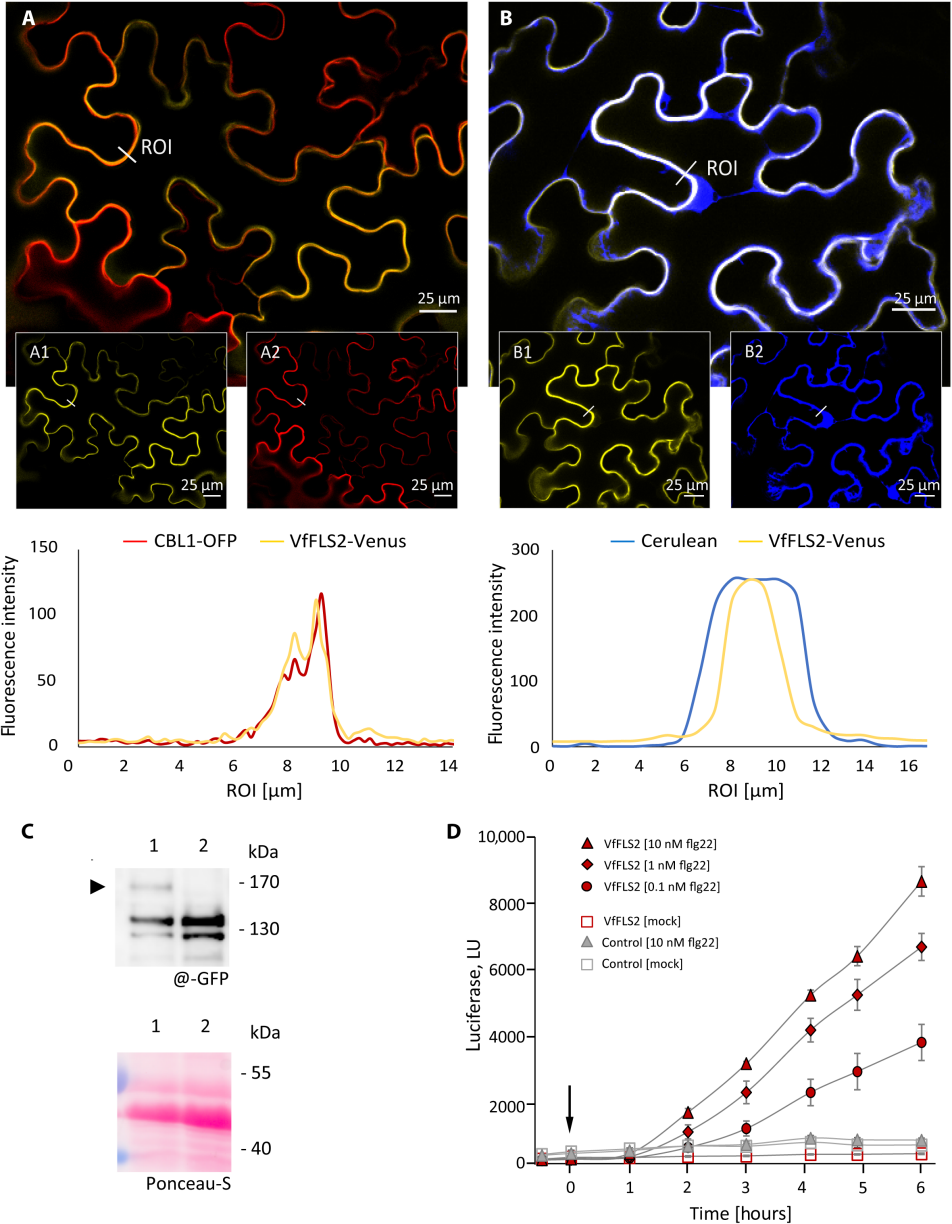
Presence, location, and functional characterization of VfFLS2. Subcellular localization of *V. faba* VfFLS2 in epidermis cells was determined via infiltration of the VfFLS2 sequence by *A. tumefaciens* in *N. benthamiana* leaves. The VfFLS2-Venus fusion protein (yellow) coexpressed with (**A**) a plasma-membrane located CBL1-OFP (red) revealed their colocalization. (**B**) Coexpression with cytoplasmatic Cerulean (blue) showed no colocalization with FLS2. The smaller pictures show the individual localization of the VfFLS2-Venus fusion protein [(A1) and (B1)] and the subcellular markers [(A2) and (B2)]. The subcellular distribution of yellow, red, and blue fluorescence was analyzed by performing a virtual line scan across a region of interest (ROI). Scale bars, 25 μm. (**C**) Western blot (top) developed with antibodies against the fluorophore tag at the C terminus of VfFLS2 indicates a protein (arrowhead) of the expected molecular mass in cotransformed protoplasts (lane 1) and not in protoplasts transformed with the reporter only (lane 2). The Ponceau-S–stained membrane is shown in the bottom to monitor similar protein loading. (**D**) Functionality of VfFLS2 was tested in mesophyll protoplasts of *A. thaliana* lacking FLS2 (*efr x fls2* mutant), cotransformed with pFRK1::luciferase, in comparison to the control transformation with the reporter gene only. Treatment of VfFLS2-expressing cells with flg22 in increasing concentrations (0.1 to 10 nM) at 0 hours (arrow) resulted in a dose-dependent increase in luciferase activity [light units (LU)] over time, confirming the capacity of VfFLS to perceive flg22 with high sensitivity and to transduce the peptide signal into a cellular response. Values and error bars indicate mean and SD of three replicates.

Phylogenetic analysis revealed a putative VfFLS2 cluster with FLS2 receptors from diverse species, including AtFLS2 (fig. S5). We characterized subcellular localization and function of a putative VfFLS2. Because of a lack of transformation protocols for *V. faba*, we transiently expressed a VfFLS2-Venus fusion construct in combination with either the plasma membrane marker Calcineurin B-Like1–orange fluorescent protein (CBL1n-OFP) or cytosol-limited Cerulean in *Nicotiana benthamiana* to verify the subcellular localization of VfFLS2. This revealed that VfFLS2, as AtFLS2 (*28*), is localized at the plasma membrane (Fig. 5, A and B).

The functionality of the VfFLS2 receptor was tested by ectopic expression in protoplasts derived from mutant *A. thaliana* plants lacking endogenous FLS2. Cotransformation of a *pFRK::luciferase* reporter [FRK (flagellin receptor-like kinase)] and the gene encoding VfFLS2 resulted in the expression of the GFP-tagged receptor (Fig. 5C), which was able to perceive the peptide ligand flg22 in a dose-dependent manner (Fig. 5D). Rising flg22 concentrations induced a higher luciferase activity due to the presence of VfFLS2 receptors.

### SE protoplasts of *V. faba* do not contain FLS2 receptors

**Fig. 6.**
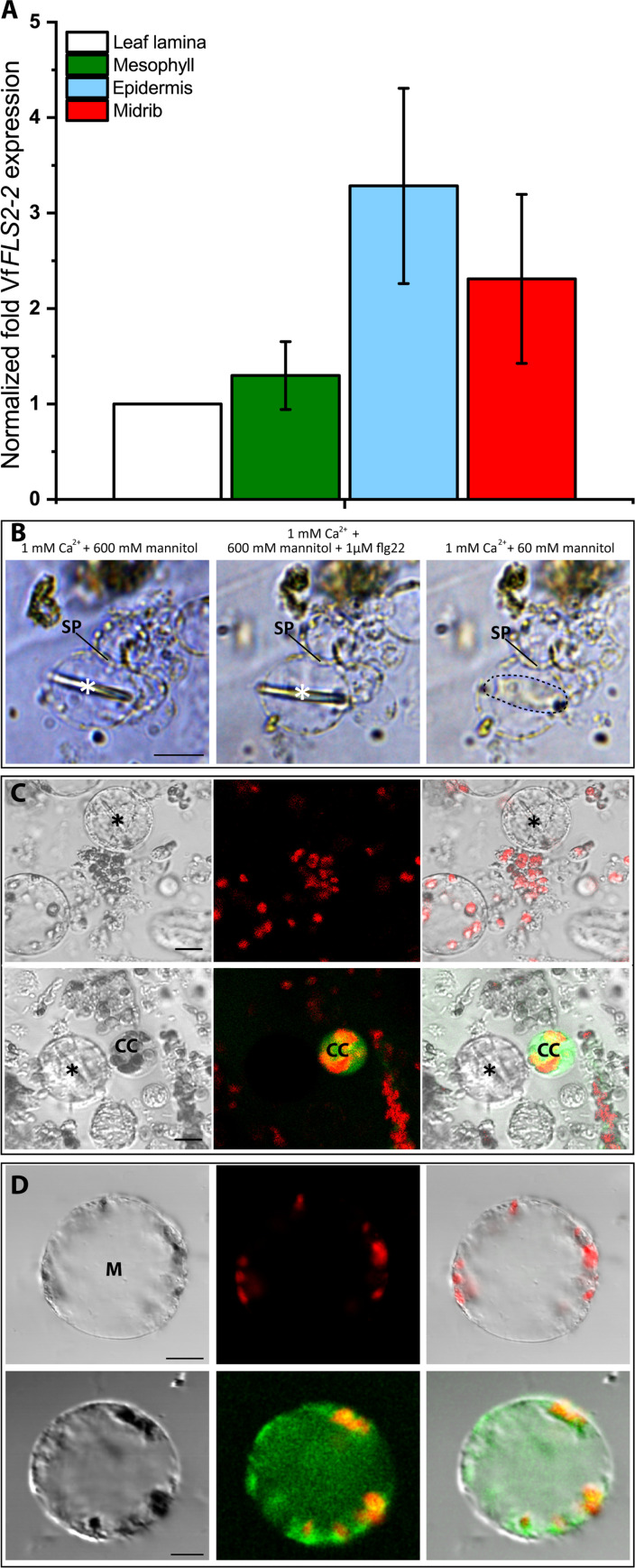
VfFLS2 receptor density in vascular cell types of *V. faba*. (**A**) The *VfFLS2* expression pattern in *V. faba* was analyzed by RT-qPCR. A tendentially higher amount of *VfFLS2* transcripts was found in the midrib and epidermal tissue as compared to the remainder of the leaf lamina. Each bar represents the mean of three technical replicates from five different plants ± SE. (**B**) The presence of FLS2 receptors in *V. faba* SE was analyzed using SE protoplasts that can be easily identified by the presence of a forisome. SE protoplasts [so-called twin protoplasts separated by a sieve plate (SP); (*35*)] were incubated in a medium containing 1 mM Ca^2+^ and 600 mM mannitol. Forisomes (asterisks) were in the condensed state and remained condensed after adding 1 μM flg22. The lacking forisome reaction to flg22 indicated an absence of FLS2 in SEs. Forisome functionality was demonstrated by subsequent perfusion of a hypoosmotic solution (1 mM Ca^2+^ and 60 mM mannitol). As a response, the protoplasts expanded and the forisomes dispersed (dashed line). Number of replicates *n* = 14. (**C** and **D**) Sensitivity for flg22 of non-SE phloem-derived protoplasts was visualized by accumulation of ROS. To that aim, 10 μM H_2_DCFDA was applied to the protoplast batch. Control protoplasts (top rows) without flg22 application showed red autofluorescence of chloroplasts in mesophyll protoplasts (M), which was absent in the larger SE protoplasts marked by a forisome (asterisks). After application of 1 μM flg22, companion cell (CC) protoplasts (C) and subepidermal/mesophyll protoplasts (D) showed a green ROS-dependent H_2_DCFDA fluorescence signal in contrast to the SE protoplasts (C) (*n* = 6) after 10 min. Transmission channel image (left), 488-nm excitation image (middle), and merged image (right). Scale bars, 10 μm

Next, it was determined whether the heterogeneous intercellular distribution of VfFLS2 receptors was similar to that of AtFLS2 receptors (Fig. 1). Comparable to the expression analysis of *AtFLS2, VfFLS2* showed a higher expression level (although not significant) in the midrib and epidermis as compared to the laminar tissue and the mesophyll (Fig. 6A; *n* = 4).

Next, we used isolated SE protoplasts to assess whether the SE plasma membrane of *V. faba* is devoid of FLS2 receptors as in *A. thaliana* (Fig. 1, E to G) despite their overwhelming presence in other vascular cells (Fig. 1). In a mixture of isolated vein cells, SE protoplasts of *V. faba* can be readily distinguished from other protoplasts by the presence of a forisome and the absence of chloroplasts. Vascular protoplasts were isolated from transport phloem strips (*35*) and immersed in an isotonic medium containing 600 mM mannitol and 1 mM Ca^2+^ (Fig. 6B). After application of 1 μM flg22, forisome dispersion was observed in none of the experiments (Fig. 6B; *n* = 14) in flagrant contrast to forisome behavior in intact plants (Fig. 8). This nonresponsiveness of forisomes in SE protoplasts may be due to either a lack of flg22 perception or the incapability to enhance Ca^2+^ influx, required for forisome dispersion (*35*).

To discriminate between these two possibilities, SE protoplasts were exposed to a hypoosmotic shock by an abrupt change from 600 to 60 mM mannitol in the external medium (*35*). As a response, the forisome inside the SE protoplasts dispersed instantaneously (Fig. 6B). This shock response is ascribed to Ca^2+^ influx following activation of Ca^2+^-permeable mechanosensitive channels (*35*). Thus, lack of FLS2 receptors rather than the inability to elevate cytoplasmic Ca^2+^ explains the nonresponsiveness to flg22 [cf. (*37*)].

Flg22 triggers a local Ca^2+^ influx coincident with ROS production in epidermal/subepidermal cells of *A. thaliana* (*14*, *38*, *39*). Therefore, the absence of FLS2 receptors in the SE plasma membrane was investigated alternatively by monitoring ROS production following flg22 application to companion cell and SE protoplasts (Fig. 6C) and mesophyll protoplasts (Fig. 6D) from *V. faba* veins. Flg22-triggered ROS production in companion cell and mesophyll protoplasts was identified using the ROS-reactive green fluorescent dye 5-(and-6)-carboxy-2′,7′-difluorodihydrofluorescein diacetate (H_2_DCFDA). Apart from punctate red spots due to chlorophyll autofluorescence, which distinguishes mesophyll protoplasts from SE protoplasts (Fig. 6, C and D), ROS-related fluorescent signals were not detected in control experiments after application of a bathing medium. Within 10 min after exposure to 1 μM flg22, green fluorescence, characteristic of ROS formation, showed up in companion cell and mesophyll protoplasts (Fig. 6, C and D; *n* = 6). In contrast, green fluorescence did not emerge in SE protoplasts in response to 1 μM flg22 (Fig. 6C; *n* = 6). These results corroborate the conclusion that the plasma membrane of SEs is devoid of VfFLS2 receptors and underscores the optical evidence for FLS2 absence in SEs as obtained with *A. thaliana* (Fig. 1, E to G).

### Flg22 triggers disparate depolarizations along the epidermis-to-SE pathway in *V. faba*

On the basis of the emergence of EPWs in vascular bundles of *A. thaliana* (Fig. 3), we postulated the transmission of an electrical signal from the epidermis to SE that precedes EPW propagation in SEs. An exact cellular allocation of the lateral voltage shift in *A. thaliana* was impossible because of the tiny dimensions of *A. thaliana* cells. The larger cells of *V. faba* enabled intracellular recordings to study whether and to which extent flg22 application depolarizes the plasma membrane of the respective cells along the presumptive signaling pathway from epidermal/subepidermal cells to SEs.

**Fig. 7.**
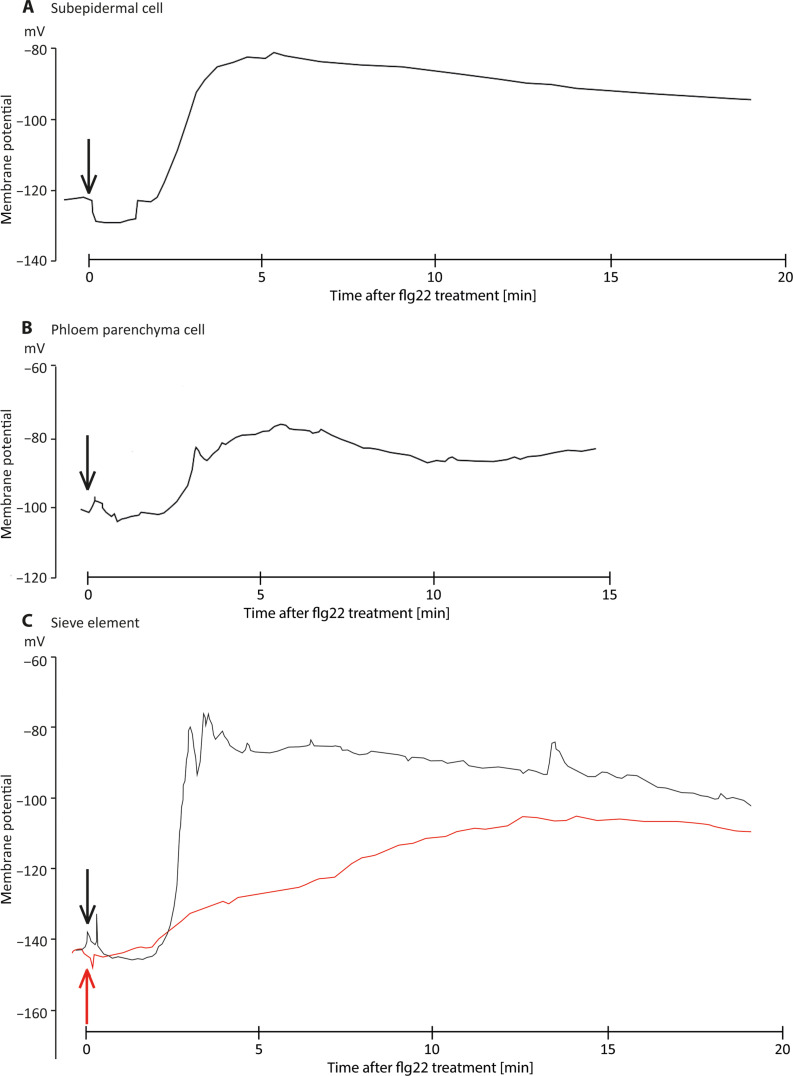
Membrane potential recordings of various cell types in *V. faba* midribs of intact leaves in response to flg22 application. The cell-specific electrophysiological response to flg22 (1 μM) was investigated through intracellular recordings in different cell types. (**A**) Subepidermal cell, (**B**) phloem parenchyma cell, and (**C**) SE. For each cell type, a depolarization was detected in response to flg22 application (black traces) but the strongest reaction was found for SEs (~65 mV). Addition of the Ca^2+^ channel blocker La^3+^ (100 μM) resulted in a strongly reduced depolarization with a flattened profile in SEs (red trace), suggesting a Ca^2+^ involvement in the flg22-induced electrical response. The time point of flg22 application is marked with an arrow. *n* = 3.

We monitored flg22-induced plasma membrane voltage shifts using microelectrodes impaled into subepidermal cells, phloem parenchyma cells, or SEs in *V. faba* veins. Flg22 application triggered voltage shifts in each cell type, albeit that the depolarization profiles strongly differed. About hundred seconds after external flg22 application (1 μM), the plasma membrane of subepidermal cells depolarized from −120 to −80 mV (Fig. 7A) and repolarized slowly to the resting level. Phloem parenchyma and SE plasma membranes depolarized ~120 s after flg22 application, exhibiting distinct kinetics. Whereas phloem parenchyma cells reacted by a small and short-lasting depolarization of 20 mV (Fig. 7B), the SE plasma membrane depolarized much more strongly from −145 to −80 mV (Fig. 7C, black trace), repolarized after 60 s to a long-lasting plateau and returned gradually to the resting level. The amplitude and duration of the plateau phase varied between the recordings (*n* = 3 biological replicates).

La^3+^, which inhibits Ca^2+^-permeable channels (*19*), inhibited forisome dispersion (*19*) and strongly reduced the flg22-induced SE depolarization phase (Fig. 7C, red trace). The data again indicate involvement of Ca^2+^-permeable channels in the flg22 response of epidermal cells, giving rise to voltage shift followed by Ca^2+^ influx throughout the cellular pathway to the SEs. There, they might be transformed into EPWs responsible for forisome dispersion and SEO in analogy to the events in the SEs of *A. thaliana*.

### Flg22 triggers forisome-based SEO in SEs of *V. faba* plants

**Fig. 8.**
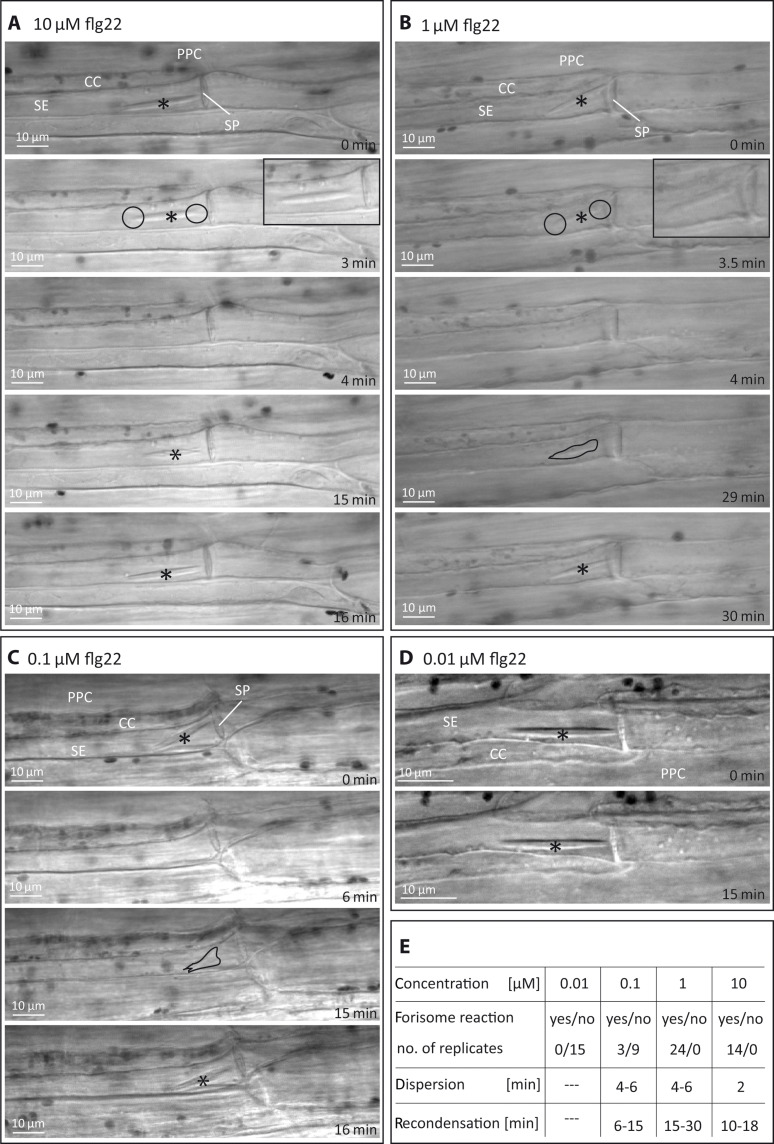
Forisome dispersion in intact SEs in the main vein of *V. faba* leaves in response to flg22 application. Forisome dispersion and recondensation in sieve tubes were monitored by successive light microscopic observations in response to local treatments with (**A**) 10 μM, (**B**) 1 μM, (**C**) 0.1 μM, or (**D**) 0.01 μM flg22 applied to individual plants. Dispersed forisomes became invisible due to a change in optical properties. [(A) and (B)] Dispersion of forisomes started at the ends (circles), subsequent to or concurrent with presumptive detachment (*21*, *40*) from the plasma membrane. Insets show a slight position change of forisomes 3 min after treatment. [(B) and (C)] Recondensation of forisomes occurred gradually. Intermediate stages are outlined in black. (D) Treatment with 0.01 μM flg22 appears to be below the threshold level required to induce forisome dispersion, whereas 1 and 10 μM are clearly above the threshold. Asterisks mark forisomes in the condensed state. SE, sieve element; SP, sieve plate; CC, companion cell; PPC, phloem parenchyma cell. (**E**) Summary of observed forisome reactions; [min] represents the time lapse after flg22 application.

To explore whether Ca^2+^ influx triggered by dose-dependent flg22 perception leads to EPW-triggered SEO by forisomes, we removed a window of subepidermal tissue from the lower side of the main vein attached to an intact plant. The exposed phloem tissue was immersed in a bathing medium (*36*) containing various concentrations of flg22, and the forisome reaction was observed by light microscopy (Fig. 8). Two minutes after application of 10 μM flg22, the forisome dispersed, and it recondensed after 10 to 18 min (Fig. 8, A and E; *n* = 14). The forisome reaction in response to 1 μM flg22 was slightly slower: The forisome dispersed after 4 to 6 min and recondensed after 15 to 30 min (Fig. 8, B and E; *n* = 24). The dispersion started invariably at the forisome ends, subsequent to presumptive detachment from the plasma membrane (*21*, *40*), and the visible phase of recondensation occurred gradually within 1 min. Application of 0.1 μM flg22 rarely induced a forisome reaction (Fig. 8, C and E; *n* = 12), and flg22 concentrations lower than 0.1 μM did not trigger forisome reactions (Fig. 8, D and E; *n* = 15). Forisome dispersion was restricted to a distance of about 0.5 cm from the site of flg22 application, which demonstrates that flg22 sensing seems to only induce occlusion of nearby SEs (fig. S6).

The behavior of *V. faba* forisomes resembles the flg22-induced transient SEO as observed in *A. thaliana*. Apparently, flg22-provoked [Ca^2+^]_cyt_ changes and SEO may be universal and show fast responses to bacterial attack. As a final note, the conformational change of forisomes in response to flg22 (Fig. 8) implicitly confirms the existence of an FLS2 receptor ortholog in *V. faba*.

### *V. faba* forisomes disperse in response to an osmotic shock

Flg22-induced forisome dispersion seems to be an effect within a limited longitudinal reach (fig. S6). We did not observe forisome dispersion more than 0.5 cm away from the site of flg22 application, suggesting that flg22-triggered SEO remains confined to the region close to the application site. An explanation for this finding might be that the electrical signal extinguishes along the pathway and becomes unable to sustain distant [Ca^2+^]_cyt_ elevations in SEs that are sufficient to trigger forisome dispersion (*33*). EPWs in SEs of *V. faba* are composed of variation potentials (VPs) and action potentials (APs), but only VPs are responsible for forisome dispersion (*33*). Distance-limited forisome dispersion along the pathway upon flg22 application suggests a fading EPW, which is characteristic of VPs (*33*, *41*). To address the exclusive involvement of VPs in SEO, forisome reactions in response to APs (*42*) induced by external glutamic acid (1 to 10 mM; *n* = 6) or γ-aminobutyric acid (GABA) (5–50 mM; *n* = 6) application were examined in planta. No forisome dispersion in response to any of these treatments was observed (fig. S7, A and B). By contrast, application of 1 M sorbitol induced a hyperosmotic shock and a subsequent forisome dispersion (fig. S7C), which indicates VP involvement.

### SEOR loss impairs phytohormone production and resistance to *Pseudomonas* in *A. thaliana*

In preceding sections, we demonstrated that bacterial attack mimicked by flg22 application induced SEO in *A. thaliana* and *V. faba* (Figs. 4 and 8), inferring that SEO may be involved in a fundamental defense response against microbial attack. Therefore, the relationship between flg22-induced SEO and the accumulation of defensive phytohormones was investigated. To that end, we compared the production and spatial distribution of JA, JA-Ile, SA, and abscisic acid in WT plants and SEO-defective mutants.

**Fig. 9.**
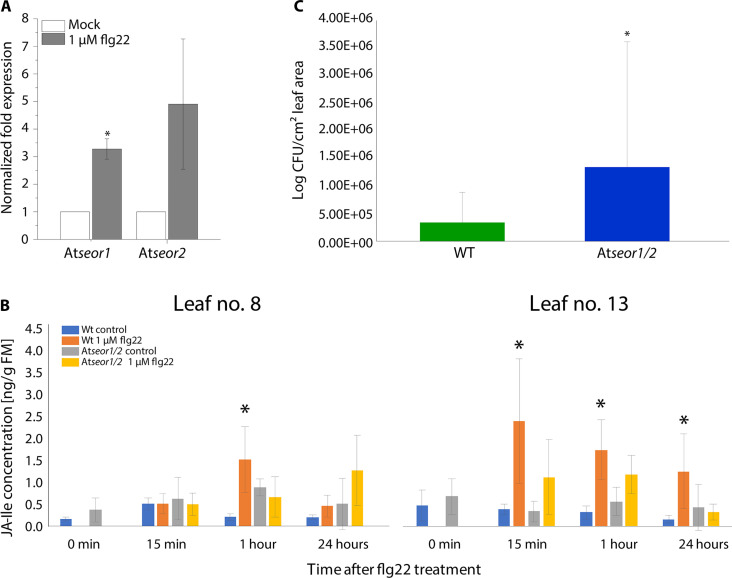
Assays related to flg22-induced suppression of Pseudomonas growth in *A. thaliana* WT (Col-0) and *Atseor*1/2 mutant plants. (**A**) Relative expression levels of *AtSEOR1* and *AtSEOR2* genes after 1 μM flg22 treatment of the whole plant. Values were normalized to the housekeeping gene (*Actin2*) and the untreated control (*n* = 4). (**B**) JA-Ile determination in *A. thaliana* WT and *Atseor1/2* double knockout plants in response to 1 μM flg22 application to leaf 8. Leaves 8 and 13 were harvested at various time points and analyzed individually with LC-MS/MS (*n* = 6 biological replicates). For all experiments, the bars represent the mean and SE of the biological replicates. The asterisks indicate statistical significance (*P* < 0.05) between the control and treatment based on Student’s *t* test. (**C**) *P. syringae* infection level in *A. thaliana* leaves 3 days after infection. Leaves of WT (Col-0) (green) and the *Atseor1/2* mutant line (blue) were infiltrated with *P. syringae* pv. tomato DC3000. Three days after the infiltration, bacteria were isolated and a dilution series is dripped out on agar plates. The infection level (log CFU/cm^2^) was determined by colony counting. The asterisk indicates statistical significance (*P* < 0.05) between WT and *Atseor1/2* mutant line based on Mann-Whitney test. WT, *n* = 40; *Atseor1/2*, *n* = 39.

In *A. thaliana*, AtSEOR1 and AtSEOR2 are the SEO proteins that operate in tandem. First evidence that flg22-induced SEO is involved in plant defense was obtained by an increased *AtSEOR1* and *AtSEOR2* expression (Fig. 9A). We determined the expression levels of both genes in the midrib of WT plants by RT-qPCR analysis 10 min after flg22 application (Fig. 9A) and found a significantly (*P* < 0.05) higher expression level for *AtSEOR1* and, although not significant, for *AtSEOR2*. To further analyze the role of AtSEOR1 and AtSEOR2 in the defense against phytopathogenic bacteria, we generated a CRISPR-Cas9–mediated double knockout line for *AtSEOR1* and *AtSEOR2*, referred to as *Atseor1/2*.

Defense-related phytohormones were analyzed upon flg22 application both in the treated local leaf 8 and the vascular-connected but untreated systemic leaf 13 (Fig. 9B and fig. S8). In leaf 8, we found that JA-Ile significantly increased after 1 hour in WT but not in the *Atseor1/2* line. After 24 hours, the JA-Ile level was still higher than in the control plants but the difference was not significant. In leaf 13, a significantly higher level of JA-Ile was detected in WT at all time points upon flg22 treatment. In the double knockout line the elevation in JA-Ile was less pronounced and not significantly different from the control after 15 min and 1 hour and absent after 24 hours (Fig. 9B). The results for JA largely mirror those of JA-Ile (fig. S8A). In contrast to JA and JA-Ile, SA was up-regulated during the first 15 min after flg22 application in the local and systemic leaf but down-regulated after 24 hours (fig. S8B). Also, in contrast to JA-Ile, WT and double mutant showed similar regulatory trends in local and systemic leaf (fig. S8B).

The responses suggest that SEO plays an integral role in the local and systemic signal transmission and is therefore likely to affect the growth of pathogenic bacteria in the plant. To test this hypothesis, bacterial proliferation was investigated. WT and *Atseor1/2* double knockout plants were locally inoculated with the pathogenic bacterium *Pseudomonas syringae* DC3000, and disease progression and bacterial proliferation were monitored after 3 days. The first five experiments (*n* = 4–12 per experiment; fig. S9) were conducted throughout the year. Despite considerable variations that were probably due to inborn season-related viability of the plants, we found a significant (*P* < 0.05) increase in colony-forming bacteria in the mutant as compared to WT plants (Fig. 9C and fig. S9). Most convincing was the negative effect of SEOR1/SEOR2 absence on bacterial resistance in the latest experiment (fig. S9, experiment 6), in which the individual heterogeneity was limited to a minimum. Together, the findings demonstrated that the absence of SEO alters defense-related phytohormone production (Fig. 9B) and increases the susceptibility to bacterial attacks (Fig. 9C).

## DISCUSSION

### Intercellular distribution and deployment of FLS2 sensors in leaf midribs

Flagellin, a filament protein of the bacterial flagellum acts as a MAMP and activates defense responses in plants and animals (*43*). After binding to the flg22 receptor FLS2, a signaling network leads to a basal defense against bacteria (*44*) by provoking several downstream defense responses to restrict pathogen growth and distribution (*1*, *44*). Flg22 perception generates local immune responses such as an increase in cytoplasmic Ca^2+^ levels (*39*) and a ROS burst (*38*).

We investigated how these flg22-triggered signals spread and transform after their generation in the epidermis along the path to the SEs and beyond. Crucial for flg22 perception is the cellular distribution of FLS2 receptors. Although *FLS2* promoter activity was found in all plant organs, and the gene is expressed in all tissue types (*14*), our confocal microscopy sections showed a heterogeneous distribution of FLS2 from surface cells toward SEs in *A. thaliana* (Fig. 1F). FLS2 receptors are strongly aggregated in epidermal cells, in particular at surfaces exposed to the environment, such as in guard cells (Fig. 1C). Within the leaf, FLS2 is sparsely present in mesophyll cells, densely deployed in a cylindrical sheath around the vascular tissues and in vascular parenchyma cells, whereas the receptor is virtually absent from SEs (Fig. 1, F and G) and, of course, xylem vessels. In *V. faba* veins, high FLS2 density was found in the epidermis and was not detectable in SEs (Fig. 6). These data suggest that the distribution of FLS2 sensors is conserved in *A. thaliana* and *V. faba*.

The absence of FLS2 receptors in SEs is consistent with the observation that *V. faba* SE protoplasts do not respond to flg22 application (Fig. 6B), whereas flg22 application to the intact plant induces SEO, probably through Ca^2+^ influx from companion cells (Figs. 2 and 8).

One wonders what the purposes of this remarkable distribution are. FLS2 receptors are abundantly deployed in epidermis cells and aggregate, in particular at the guard cell surface (Fig. 1). As an advanced post of bacterial recognition, stomata are thus heavily lined with perception sites along the path giving easy access to bacterial intruders. The deployment of FLS2 in the vascular sheath seems less logical. However, numerous animal pathogens only inflict damage to the softer-outer leaf tissues (*45*), not to the more rigid vascular cells encapsulated by a, probably stiff, cylindrical sheath, which is directly exposed to bacterial infection in such instances. Hence, it is tempting to assume that the FLS2 receptors located in vascular cells, particularly in the vein sheath, form a second line of bacterial recognition after surface damage. As a further speculation, the absence of FLS2 sensors in SEs would prevent a confusing interference between FLS2-induced Ca^2+^ influx and Ca^2+^ influx associated with systemic EPWs.

### Flg22 sensing triggers biphasic Ca^2+^ influx into the cytosol mediated by several types of Ca^2+^-permeable channels

Upon flg22 application, a Ca^2+^ influx emerged after ~1 min, followed by a second peak after ~3 min (Fig. 2, B and C). A cellular Ca^2+^ influx is mediated by Ca^2+^-permeable channels located in the plasma membrane. Thor *et al.* (*29*) showed an activation of the *A. thaliana* Ca^2+^-permeable channel OSCA1.3 via phosphorylation in response to flg22. This Ca^2+^ elevation may trigger gating of Ca^2+^-activated Ca^2+^ channels located in the endomembrane system, such as the endoplasmic reticulum (ER) (*46*). Like in animals, the ER not only may serve as a Ca^2+^ store determining the generation of [Ca^2+^]_cyt_ waves [e.g., (*47*)] but also acts as a sink for Ca^2+^ in plants (*48*). The collective and concerted action of these channels may increase the [Ca^2+^]_cyt_ level to an extent, leading to vigorous transmembrane ion exchange. Supporting the interplay of Ca^2+^ in different membranes, pharmacological analyses indicate that the flg22-triggered Ca^2+^ response in guard cells depends on Ca^2+^ influx and Ca^2+^ release from internal stores (*39*). The turgor collapse resulting from the initial rise of Ca^2+^ concentration may lead to the activation of mechanosensitive Ca^2+^ channels (fig. S10). Successive involvement of several Ca^2+^-permeable channels may thus lead to a dual voltage shift. Admittedly, the evidence in favor of the successive influx is fragmentary and thus highly speculative but seems to fit with the observations thus far. The obvious relevance of a turgor collapse (*49*) in response to flg22 by guard cells appears to be that the stomata are closed (*50*) so that the microbial invasion is halted.

### Pathway of the two-component voltage shift rolling from the epidermis to SEs

The presumptive order of events suggests that the biphasic Ca^2+^ influx profile (Fig. 2, B and C) leads to dual cell-to-cell voltage shifts, mediated by voltage-activated and mechanosensitive Ca^2+^-permeable channels, respectively. The voltage disbalances can be passed on to adjacent cells via plasmodesmata that present the sites of lowest electrical resistance (*51*). Truncation of plasmodesmata permits the “lifestyle” of guard cells to be essentially different from that of the bordering cells (*52*). Furthermore, an absence of plasmodesmal contacts will virtually impede electrical traffic between guard cells and their neighbors (*52*) and limits an flg22-induced turgor collapse to the guard cells without further dissemination of a turgor relaxation.

By contrast, the epidermal pavement cells have abundant symplasmic contacts to adjacent cells (*53*). Their symplasmic connectivity allows ample propagation of voltage shifts to neighboring cells. In *A. thaliana*, the retarded Ca^2+^ influx into vascular cells as compared to the lamina cells (fig. S1) is a first indication of a voltage shift rolling from the epidermis to SEs. Likewise, extracellular electrophysiological measurements show the passage of a hyperpolarization wave toward the SEs and along the SEs in *A. thaliana* (Fig. 3). Moreover, intracellular electrophysiology of successive cells demonstrates a depolarization wave that clearly moves from the epidermis to SEs in *V. faba* veins (Fig. 7).

It should be stressed that the symplasmic connectivity is not persistent: After the rise in intercellular Ca^2+^ concentration, plasmodesmata are probably closed due to Ca^2+^-induced callose constriction (*23*). The Ca^2+^ wave of the voltage shift induces closure of plasmodesmata as shown in many studies [e.g., (*54*, *55*)]. The symplasmic corridor will remain closed until excess cytosolic Ca^2+^ is removed (*21*). All in all, the conclusion inferred by Ca^2+^ visualization (Fig. 2) and electrophysiology (Fig. 3) is that depolarization at the recognition surfaces, initiated by Ca^2+^ influx (*2*, *56*), triggers a voltage shift that is transmitted via successive cells toward the SEs (fig. S11).

### The nature of the dual voltage shift

**Fig. 10.**
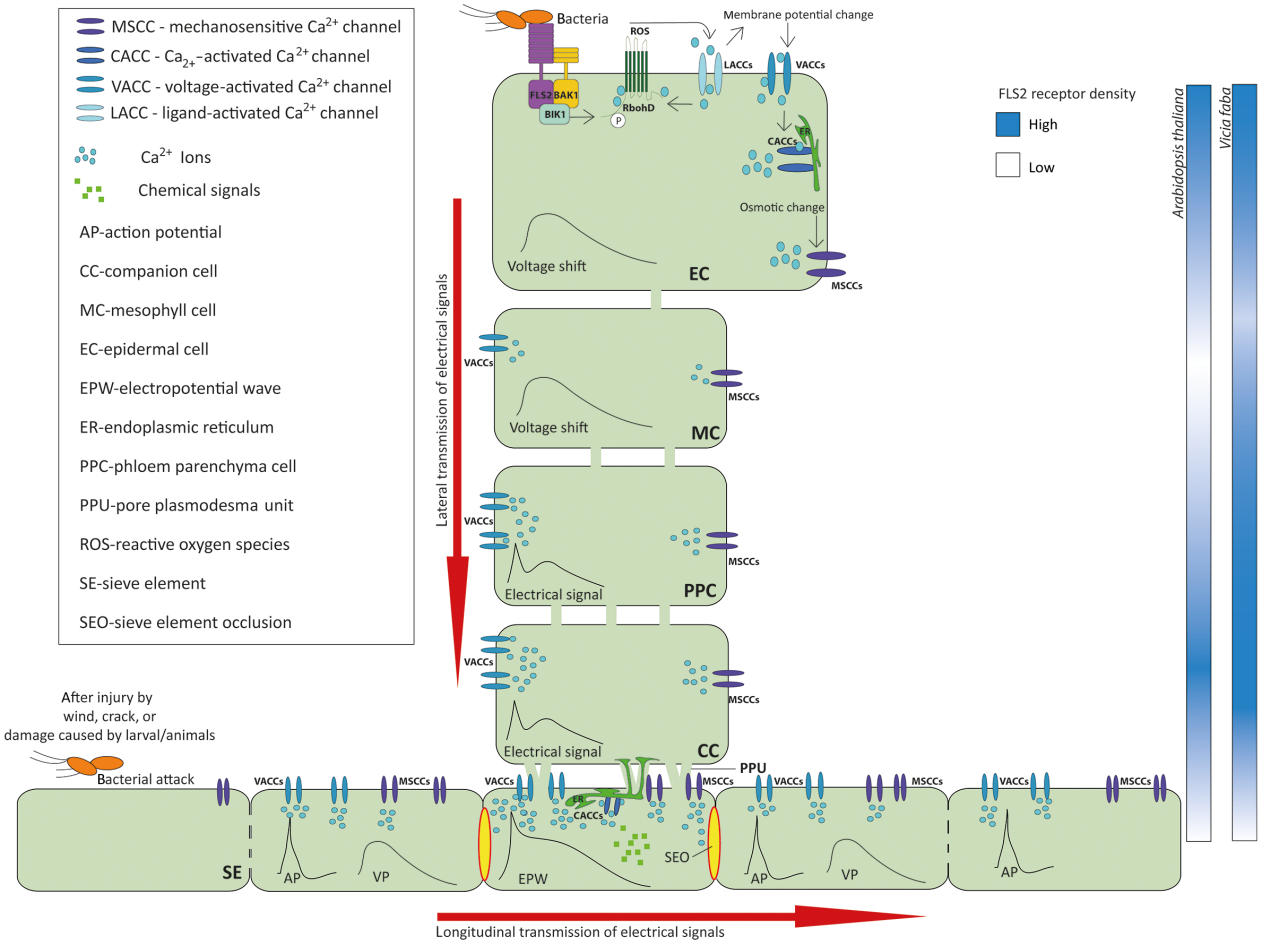
SEO effected by VPs triggered by flg22 application. Bacterial flg22 is sensed by aggregates of FLS2 receptors located in the plasma membrane of epidermal and subepidermal cells [cf. (*14*)] but also in that of the phloem parenchyma cells and companion cells (Fig. 1), whereas the SE plasma membrane is devoid of FLS2 receptors (Figs. 1 and 6). In FLS2-rich cells, flg22 binding induces a Ca^2+^ influx from the apoplast into the cell interior via Ca^2+^-permeable channels (see fig. S10). The resulting intracellular rise of Ca^2+^ triggers a depolarization of the plasma membrane and ROS production (*26*). The dual voltage response probably initiates trafficking of two successive overlapping voltage shifts via the plasmodesmata, giving rise to APs and VPs propagating via the SEs. The amount of Ca^2+^ released into SEs by VPs affects forisomes, giant protein bodies in legume SEs, which act as sieve tube plugs after wounding or pathogenic attacks (*19*, *21*). Swelling or dispersion of forisomes is Ca^2+^ dose dependent, and hence, they may react to the Ca^2+^ elevation during EPW passage in SEs. Thus, the forisome reaction serves as an indicator for massive Ca^2+^ influx. Forisomes are insensitive to APs (*33*), which demonstrates that Ca^2+^ influx mediated by APs falls short to meet the dispersion threshold and that only VP-mediated Ca^2+^ influx exceeds the dispersion threshold. VPs provoke a transient closure of SEs and, probably, the plasmodesmal connectivity of nearby vascular cells. In this way, a temporary insulation of the area in question is reached, which may contribute to mounting defense mechanisms.

The voltage shift rolling from the epidermis to SEs is composed of two components (fig. S11), which likely corresponds with a biphasic Ca^2+^ influx in the cells along this path. The peaks are probably related to the quick and slower Ca^2+^ influx and associated depolarizations in flg22-treated cells (Figs. 3, 7, and 10). In analogy to the events in epidermal cells, the first component likely depends on the engagement of voltage-activated Ca^2+^-permeable channels and the second on the gating of mechanosensitive Ca^2+^-permeable channels. Notably, the second component that may triggers forisome dispersion is much more susceptible to the flg22 concentration (Fig. 8). This dose-response behavior is in line with the idea that the second component is turgor dependent because the Ca^2+^ influx increases with the flg22 concentrations due to a mounting loss of intracellular ions (*56*).

### Transformation of an EPW into a long-distance electrical signal

In animal sensory organs, a crucial event in electrical long-distance signaling is the transformation of a relatively slow intercellular electrotonic transmission into a rapid longitudinal transmission via the neurons. Likewise, the local electrical signal generated at flg22-perceiving surfaces could be transformed into a long-distance signal upon arrival in the SEs. As a matter of fact, we observed an exceptional depolarization in SEs (Fig. 7), which suggests that the electrical signal is amplified and transformed at the companion cells by shaping the function of the *GLR* genes *GLR3.3* and *GLR3.6* upon arrival in SEs (*57*). This could be facilitated by a high density of Ca^2+^-permeable channels, located in the SE plasma membrane (*19*). In addition, the lower electrical resistance of sieve pores as compared to plasmodesmata will allow a faster propagation of electrical signals through SEs. It should be underscored once more that the voltage response of the FLS2-devoid SEs to flg22 application (Fig. 7) essentially depends on the flg22-sensing in non-SE cells. They generate the depolarization, which is transmitted to SEs and amplified there.

Given the dual character of the voltage shifts arriving at the SEs, it is no surprise that the biphasic signals are amplified and converted to two merged or sequential EPWs [e.g., (*20*)] initiated by tidal waves of Ca^2+^ influx along the SE path. As in the intercellular pathway from the epidermis to SEs, the first peak (AP) would be associated with the operation of voltage-activated channels and the second one (VP) with gating of mechanosensitive Ca^2+^-permeable channels along the sieve tube [e.g., (*21*, *58*)]. Such a dual depolarization pattern was also observed for EPWs in SEs after burning *V. faba* leaf tips (*19*, *23*, *33*).

Although the character of both EPWs seems almost identical at first sight, the origin and nature of APs and VPs are quite different, which is crucial for interpretation of the present results. APs depend on electrical propagation along domino arrays of voltage-activated Ca^2+^-permeable channels deployed in the SE plasma membrane, whereas VPs primarily depend on turgor gradients sensed by mechanosensitive Ca^2+^-permeable channels in cells flanking SEs and in the SEs themselves (*21*). APs are more rapid, have a low Ca^2+^ influx capacity, and can propagate almost infinitely throughout plants, whereas VPs are slower, have a high Ca^2+^ influx capacity, and have a limited longitudinal reach due to progressive decline of turgor effects along the pathway (*21*). The differences in Ca^2+^ influx capacity and in longitudinal reach between the APs and VPs are of crucial importance for interpretation of the events.

### Impact of Ca^2+^ influx on dose-dependent responses of sieve pore occlusion and constriction

Studies in *Cucurbita maxima* and *V. faba* demonstrated that sieve pore closure is a dual mechanism (*19*, *23*, *24*): Instant occlusion by proteins (*19*, *23*) is followed by a slower sieve pore constriction by callose deposition. Ca^2+^-mediated sieve pore plugging in legumes depends on Ca^2+^ binding to aspartate residues of SEO proteins, the highly ordered building blocks of forisomes [e.g., (*59*)]. Their molecular reorganization causes forisome dispersion and swelling so that the sieve pores becomes occluded (*36*, *37*).

Callose synthesis depends on Ca^2+^ binding to a transmembrane CalS complex that delivers callose at the outside face of the plasma membrane so that the sieve pore becomes constricted. Callose deposition requires the Ca^2+^-binding protein CALMODULIN-LIKE 41 (CML41), whose expression is up-regulated by flg22. CML41 facilitates callose deposition at plasmodesmata 30 min after flg22 treatment (*60*). Sieve pore plugging and constriction are transient because forisomes recontract (*37*) and callose is degraded [e.g., (*23*)] after removal of excess Ca^2+^.

Sieve pore plugging and constriction thus both depend on cytosolic Ca^2+^ concentration in SEs albeit that the threshold for forisome dispersion appears lower than that for callose synthesis (*21*). Ca^2+^ ions for elevation of the cytosolic Ca^2+^ pool in SEs are recruited from cell walls, and ER stacks appressed to the SE plasma membrane (*19*) by gating of voltage-dependent and/or mechanosensitive Ca^2+^-permeable channels and Ca^2+^-modulated channels, respectively (*19*, *21*). Forisome dispersion is only provoked by VPs and not by APs, which indicates that only Ca^2+^ influx elicited by VPs is sufficient to meet the threshold for forisome dispersion (*33*). In *A. thaliana*, this systemic Ca^2+^ response to turgor pressure changes in the phloem relies on the activation of glutamate receptor-like channels (*61*).

### The VP is the EPW engaged in SEO

It is not entirely certain whether SEO in *A. thaliana* depends on plugging mediated by SEOR proteins because good evidence shows that the unordered AtSEOR1/AtSEOR2 conglomerates are unable of plugging in intact plants (*62*). However, this is not true for damaged (*63*, *64*), and phytoplasma-infected (*65*) or Pseudomonas-mimicked (Fig. 4) *A. thaliana* plants. Yet, *V. faba* plants are much more suitable for assessment of closure events owing to the unequivocal and visibly discrete reactions of large SEO protein constructs such as forisomes.

As shown in vitro (*37*) and in vivo (*19*), forisome dispersion is Ca^2+^ dose dependent. Thus, a Ca^2+^ threshold is to be surpassed in SEs to trigger forisome dispersion. Usually, only VPs are able to do so (*33*). Thus [Ca^2+^]_cyt_ elevations associated with VPs appear to meet the dispersion threshold, whereas those elicited by APs do not, which may indicate that mechanosensitive channels believed to mediate VPs are quantitatively more effective for Ca^2+^ influx (*58*). Several arguments support a principal role of VPs in forisome dispersion: (i) Application of GABA or glutamic acid, which both induce APs [e.g., (*20*)], did not induce forisome dispersion (fig. S7). (ii) By contrast, VPs triggered by burning leaf tips of *V. faba* were responsible for forisome dispersion (*19*, *33*). (iii) Application of flg22 strongly stimulates the second voltage shift (Fig. 7) that is presumably related to gating of mechanosensitive channels and, hence, to VPs. (iv) Furthermore, sieve pores become occluded above 1 μM flg22, when the VP may be vigorous enough for sufficient Ca^2+^ influx. (v) In line with the usual fading behavior of VPs (*20*, *22*), the effect of flg22 on forisome dispersion is distance limited, i.e., the flg22 response is only detectable within a few centimeters away from the application site (Fig. 8 and fig. S6). The flg22 effect may occur in a dome-like area of decreasing turgor loss extending from the site of flg22 application. Beyond the dome (fig. S11), Ca^2+^ influx may be insufficient to meet the threshold for forisome dispersion.

As a final note regarding the occlusion events, it is difficult to understand how CF can be removed by mass flow from sieve tubes behind the barricade after sieve pore occlusion (Fig. 4). A plausible explanation, however, is that, because sieve tubes are only occluded over a limited distance, SEs behind the barrier start attracting water and restore mass flow from this point so that CF is cleared out from the SEs behind the barrier. As soon as the barrier is lifted after Ca^2+^ removal, additional CF will then enter the reopened SEs (Fig. 4).

### Functional potential of an flg22-triggered EPW

Long-distance signaling via sieve tubes plays a major part in the response of plants to mechanical damage [e.g., (*21*, *58*)]. Systemic signaling of wounding is commonly ascribed to EPWs and Ca^2+^ waves, which induce the production of JA and its subsequent release into SEs (*12*, *58*, *66*). Now, the jasmonate-based response of *C. maxima* to flg22 application (*12*) and the reaction to flg22 application in *Arabidopsis* (Figs. 2, 3, 4, 7, and 8) fit well with the idea that biotic stresses can also elicit EPWs (*12*).

It seems reasonable to assume that flg22-sensed bacterial attacks elicit APs and VPs in tandem (Fig. 10 and fig. S11). Undoubtedly, Ca^2+^ ions play a pivotal role in electrical and chemical long-distance signaling (*19*, *25*, *42*). In contrast to animals, EPWs in plants are not primarily intended to convey electric messages but rather confer ion redistribution between the cytoplasm, organelles, and cell walls along the propagation pathway. By consequence, EPWs in plants are intended to boost Ca^2+^ influx and induce inherent changes in Ca^2+^ signatures in distant cells as a prime goal (*21*, *25*).

APs and VPs probably experience a limited reciprocal interference. As APs are faster and unable to barricade the pathway (*33*), the propagation pathway of VPs is not shut by precedent APs.

### Speculations on transient symplasmic reorganization along the VP propagation pathway

The symplasmic reorganization associated with the passage of VPs was speculated to grant an increased autonomy to the insulated cells for a limited period of time (*21*). This hypothesis urges a more detailed examination of the events involved in SEO and associate plasmodesmal closure. Passage of a VP induces a closure of sieve pores (*19*, *23*), which is reversed within 15 to 60 min (*19*, *23*, *24*). This lifting of the sieve pore barriers may occur upon Ca^2+^ removal by pumps (P-type Ca^2+^ adenosine triphosphatase) and CAX or BICAT family antiporters (*46*).

A turgor collapse in the cells flanking the SEs and beyond induces Ca^2+^ influx and resultant plasmodesmal closure. Moreover, the Ca^2+^ influx may induce plasmodesmal closure so that the cells gain autonomy due to impairment of the symplasmic connectivity. During the transient symplasmic insulation, modified Ca^2+^ signatures may trigger signal cascades for immune responses that are normally controlled and suppressed by symplasmic interaction (*21*). In this way, chemical signals are produced that are released in the phloem stream after clearance of the symplasmic barriers. Among solutes that are able to travel long-distance, oxylipins, a glycerol-3-phosphate dependent signal, the lipid transfer protein DEFECTIVE IN INDUCED RESISTANCE1, azelaic acid, dehydroabietinal, and *N*-hydroxypipecolic acid were proposed as systemic information carriers [reviewed in (*67*)].

The key question now is whether SEO (as the most manifest sign of transient symplasmic insulation) in response to flg22 application participates in the plant defense system. In other words, is SEO related to an enhanced production of phytohormones such as JA and SA that confer resistance to bacterial attacks?

### Do VP-associated SEO and vascular reorganization participate in systemic signaling for increased resistance?

To address the above question, phytohormone synthesis in response to flg22 application was compared between WT plants (capable of SEO) and *Atseor1/2* knockout mutants (incapable of SEO) (Fig. 9). JA and JA-Ile concentrations were significantly more up-regulated in the flg22-treated and in the nontreated leaf of WT plants compared to the mutants (Fig. 9). However, the fact that JA and JA-Ile appear earlier in the distant leaf 13 than in the treated leaf 8 is somehow unexpected. It should be borne in mind, however, that systemic wound signals travel very fast (minutes) and cause an almost instant outburst of JA synthesis in systemic leaves. After 30 min, the JA level of a wounded *Arabidopsis* leaf causes an increase in JA in systemic leaves that is half of the elevation in wounded leaves. The level further increases in wounded leaves but quickly declines in systemic leaves after this time point (*68*). Our experience from wounding experiments let us expect that, in systemic leaves, jasmonates do not show up earlier than in the treated leaves (*69*). It is quite thinkable that, at lower time lapses after treatment (like 15 min in our experiments), the ratio of JA synthesis further shifts toward the systemic leaves. It could well be that flg22 spraying benefits JA synthesis in systemic leaves rather than wounding for as yet unknown reasons. This implies that SEO effectuated by flg22-induced VPs must play a substantial part in enhancement of the plant resistance against bacterial infection. At this stage, it is unclear how Ca^2+^ influx and symplasmic insulation of SEs and flanking cells modify the metabolic cascades responsible for JA synthesis and how relevant a putative transient cell autonomy is for increased JA synthesis (*21*).

Over 3 days, WT *A. thaliana* plants show a higher resistance against Pseudomonas infection than *Atseor1/2* mutants (Fig. 9B), which is unlikely to be assigned to physical constraints given the transient character of the SEO (Figs. 4 and 8). It therefore corroborates the fact that SEO and associated events play a pivotal role in mounting increased resistance against bacterial infection.

All in all, the present results reveal an unexpected VP involvement in bacterial resistance as a result of flg22 perception. They also add unique elements to the “classic view” that wounding is responsible for JA synthesis and signaling: Bacterial infection also induces JA messaging. It was demonstrated, furthermore, that transient SEO and an associated symplasmic reorganization is a crucial event in increased JA synthesis and signaling as a response to flg22 application.

## MATERIALS AND METHODS

### Plant material

For microscopic analyses, electrophysiology, Ca^2+^ measurements with the photon-counting camera system, and pathogen assays, plants of *A. thaliana* (Col-0, *fls2* mutant Sail_691C4, *pFLS2::FLS2*-3xmyc-GFP, pMAQ2, KC274, *Atgsl7*, and *Atseor1/2*) and *V. faba* cv. Witkiem major (Nunhems Zaden) were cultivated for 5 weeks in pots in a greenhouse at 20° to 30°C, 60 to 70% humidity, and a 14-hour/10-hour light/dark regime. Supplementary lighting (SONT Agro 400 W; Philips) gave an irradiance range of 200 to 250 mmol^−2^ s^−1^ at the plant apex.

For Ca^2+^ measurements in the luminometer and analysis by RT-qPCR, *A. thaliana* plants were used 14 days after germination on Hoagland (HL) medium (*70*) vertically grown under a 16-hour/8-hour light/dark regime and a light intensity of 80 μmol m^−2^ s^−1^ at 23°C. The *A. thaliana* pMAQ2 line, expressing cytosolic apoaequorin (kindly provided by M. Knight), the vascular cell–specific GAL4 enhancer trap line KC274 (*32*), and Aeq^cyt^*fls2* (kindly provided by J. Lee) were used for these measurements.

For the subcellular localization of the *V. faba* VfFLS2 protein, *N. benthamiana* plants were grown in soil under longday (LD) conditions (16-hour light; 8-hour darkness) in the greenhouse at 22° to 25°C in the light and 19° to 25°C in the dark. Artificial light was switched on when the natural light fell below 700 μmol m^−2^ s^−1^.

For the Pseudomonas infection assay, *A. thaliana* plants (Col-0 and *Atseor1/2*) were grown in a growth chamber under constant temperature (23°C) and a day/night rhythm of 16-hour light with an artificial light of 100 μmol m^−2^ s^−1^ and 8-hour darkness. Plants were cultivated under short-day conditions (8-hour light; 16-hour darkness). For bacterial infection, 5- to 6-week-old plants were used.

### Visualization and localization of FLS2 receptors in Arabidopsis midribs by CLSM

To localize the FLS2 receptor density in different cell types, the FLS2-GFP reporter line *pFLS2::FLS2*-3xmyc-GFP was used (kindly provided by C. Zipfel) (*28*). Longitudinal sections of the leaf mid rib of 5-week-old plants were sliced by hand with a razor blade and placed with a droplet of water on an objective slide. Samples were examined using an LSM 880 microscope (Carl Zeiss, Germany) with the 488-nm laser line produced by an argon multiline laser (11.5 mW). Images were taken by a 40x objective (Plan-Apochromat 40_/0.8). Lambda stacks were created using the 32-channel GaAsP detector followed by linear unmixing with the ZEN software. *Z*-stacks were taken from relevant areas of the samples, and maximum intensity projections were produced with the ZEN software.

To enhance the signal intensity, the background fluorescence of the sections was reduced by incubation in ClearSee solution (*71*), followed by fluorescence enhancement through antibody staining against GFP, as described by Schnieder *et al.* (*72*). The longitudinal sections were fixed with 4% (v/v) formaldehyde in phosphate-buffered saline (PBS). Vacuum was applied (3 x 5 min) until the sections stopped floating. After 1-hour incubation, the sections were washed in PBS (3 x 5 min) and transferred to ClearSee solution [xylitol (10% w/v), sodium deoxycholate (15% w/v), and urea (25% w/v) in H_2_O] and incubated overnight at room temperature with gentle agitation. The sections were then washed (PBS; 3 x 5 min) and transferred to blocking buffer (PBS containing 5% nonfat dry milk) for 3 hours at 4°C, followed by washing (PBS; 3 x 5 min). Subsequently, the sections were incubated in blocking buffer containing primary GFP Living Colors A.v. monoclonal antibody (JL-8) [#632381; TaKaRa (Clontech Laboratories Inc., Montain View, Canada) (diluted 1:1000] overnight at 4°C. The sections were then washed (PBS; 3 x 5 min) and incubated in blocking buffer containing Alexa Fluor 488, goat anti-mouse IgG (H+L) cross-adsorbed secondary antibody (A-11001, Thermo Fisher Scientific (Waltham, USA) (diluted 1:1000) for 3 hours at room temperature. After a wash step (PBS; 3 x 5 min), the sections were incubated in ClearSee solution containing Fluorescent Brightener 28 (0.1 mg/ml) to visualize the cell walls and sieve plates. The sections were analyzed on a CLSM (Stellaris 8 Falcon, Leica Microsystems GmbH Wetzlar, Germany) using excitation/emission wavelengths of 491/500 to 555 nm for the antibody and 405/440 to 460 nm for the cell walls.

### Ca^2+^ measurements by aequorin luminescence imaging in *A. thaliana*

#### Cytosolic free Ca^2+^ in leaves

For visualization of [Ca^2+^]_cyt_, a high-resolution photon-counting camera system (HRPCS218, Photek, St. Leonards on Sea, UK), consisting of an intensified charge-coupled device camera (ICCD218; Photek) and a camera controller (HRPCS4; Photek) was used. The camera was mounted on a dark box (DB-2; Photek). Whole plants were sprayed with 10 μM coelenterazine (native CTZ, P.J.K. GmbH, Kleinblittersdorf, Germany) dissolved in 0.01% Tween 20 and incubated overnight in the dark. Furthermore, single leaves of intact 5-week-old *A. thaliana* plants (line pMAQ2) were analyzed in the HRPCS218 system after spraying leaves with either 1 μM flg22 (Davids Biotechnologie GmbH, Regensburg, Germany) or water as a control. Photons were captured in the photon-counting mode with a 200-ms frame rate, and 300-s cumulative images were integrated offline after the experiments. After each treatment, discharge of the remaining aequorin was recorded by application of 1 M CaCl_2_ in 10% ethanol to the whole rosette. Luminescence values were normalized by calculating *L*/*L*_max_ (luminescence counts per s/total luminescence counts remaining) as described previously (*31*).

#### Cytosolic Ca^2+^ in whole seedlings and leaf sections

Cytosolic free Ca^2+^ elevation was studied with the pMAQ2, KC274, and Aeq^cyt^*fls2* lines in a Luminoskan Ascent luminometer (Thermo Electron Corporation, Finland). Aequorin was reconstituted overnight in the dark by incubating 14-day-old whole seedlings of the *Arabidopsis* lines or excised midribs/lamina of pMAQ2 with 10 μM coelenterazine in 0.01% Tween 20 in 96-well plates. Following luminescence background determination for 1 min, 50 μl of flg22 was added to each well to a final concentration of 10, 100, or 1000 nM (control plants were treated with distilled water), and the luminescence signals of the whole seedlings were recorded for 13 min according to Johnson *et al.* (*70*). For more detailed analysis, the standard measurement parameter of 6-s measuring interval was reduced to 4 s (fig. S1). After each treatment, discharge of the remaining aequorin was recorded by application of 1 M CaCl_2_ in 10% ethanol to each well. [Ca^2+^]_cyt_ was calculated from the obtained relative luminescence units (*70*).

### Extracellular electrophysiological measurements in *A. thaliana*

*A. thaliana* plants were grown on half-strength MS media [MS salts (2.15 g/liter), MES (0.5 g/liter), sucrose (13.7 g/liter), and Gelrite (3 g/liter); pH = 5.8] and transferred after 21 to 24 days into large glass petri dishes filled with soil (Substrat 2, Klasmann-Deilmann, Germany). Five-week-old plants were installed for electrophysiology to prevent electrical disturbances and, hence, guarantee stable electrical recordings. Before the measurements, the soil was covered with a plastic foil to impede contacts between the wetted soil and leaves, each single leaf was electrically isolated using small plastic foil strips, and the upper side of the leaf to be investigated was carefully fixed with double-adhesive tape on a glass microscope slide. According to Zimmermann *et al.* (*22*), the tips of two electrodes were pierced blindly into the lower side of the main vein. The reference electrode was placed into the soil.

Using two application glass capillaries (Hilgenberg, Germany), flg22/buffer solution or apoplasmic buffer solution [2 mM KCl, 1 mM MgCl_2_, 1 mM CaCl_2_, 50 mM mannitol, and 2.5 mM MES with a pH of 5.7; with 0.1% Tween 20 (v/v)] as a control was added to the lower leaf tip at a distance of 2 cm from the measuring electrodes. The glass capillaries were connected to 50-ml syringes fit to press solutions from the glass capillaries onto the leaf epidermal tissue without mechanical disturbance of the electrodes.

Electrophysiological measurements started with a resting period of 2 to 4 hours to achieve a stable voltage baseline. Then, a drop (10 μl) of buffer solution was administered onto the leaf epidermal tissue as a negative control. After 1 hour, the flg22 solution (10 μl) was supplied to the leaf surface. When the flg22-induced voltage wave had been finished after 1 to 2 hours, proper functioning of the setup was checked by breathing. CO_2_ enrichment of the air by breathing near the plant evokes a typical electrophysiological reaction (*73*).

Positioning of the measuring microelectrodes and application microcapillaries with the aid of micromanipulators (model ST 35; Brinkmann Instrumentenbau) and solute application were optically surveyed with a stereomicroscope (Stemi 305, Zeiss, Germany). Voltage shifts were determined with an amplifier (Dual Microprobe System Model KS-700, World Precision Instruments, Sarasota, USA) and captured by an analog chart recorder (W+W Recorder Model 314).

### Visualization of SE occlusion in *A. thaliana* by CLSM and fluorescence microscopy

Flg22-induced SEO in *A. thaliana* was studied after application of the phloem-mobile fluorochrome CFDA mixed isomers (Invitrogen, Karlsruhe, Germany). A CFDA stock solution was prepared (1 mg CFDA in 1 ml of dimethyl sulfoxide). Before loading sieve tubes with CFDA, 1 μl of CFDA stock solution was dissolved in a 2-ml bathing medium [2 mM KCl, 1 mM CaCl_2_, 1 mM MgCl_2_, 50 mM mannitol, and 2.5 mM MES/NaOH buffer (pH 5.7)]. A cropped leaf tip (~0.2 cm) of an intact plant was dipped into the CFDA solution. CFDA permeates the plasma membrane in the nonfluorescent acetate form and is cleaved in the cytosol to the membrane-impermeable fluorescent compound CF. The trapped CF is transported inside SEs by mass flow and observed by CLSM or fluorescence microscopy (*36*).

After ~2 hours of CFDA loading, 100 μl of flg22 (1, 10, and 100 nM and 1 μM; Davids Biotechnologie, Regensburg, Germany) solved in a bathing medium or 100-μl pure bathing medium as a control was pressure infiltrated into the leaf epidermal apoplast left and right from the midrib by a carefully installed 1-ml syringe. To visualize mass flow in sieve tubes, freehand cross sections of the midribs of fresh unfixed leaves were made 2 cm above and 2 cm below the infiltration site at fixed time points (10, 90, 180, and 240 min) after infiltration. CLSM observations were made at the 488-nm line of a CLSM (SP2, Leica Microsystems, Heidelberg, Germany). The experiment was repeated 15 to 33 times for each flg22 concentration with WT, *Atseor1/2*, *gsl7*, and *fls2* plants. Fluorescence was observed using a GFP filter block (BP 470/40, FT 495, and BP 525/50; Carl Zeiss, Göttingen, Germany) of a fluorescence microscope (AxioImager.M2, Carl Zeiss, Göttingen, Germany).

### Evolutionary relations of FLS2 with reference to VfFLS2

Domain searches were made with PROSITE (http://expasy.ch/prosite), whereas leucine-rich repeat (LRR) receptor motifs were searched using the MEME software (*74*). To perform a phylogenetic analysis, the protein sequence of putative VfFLS2 was compared to the protein sequences of AtFLS2 and other 20 *Arabidopsis* members of the LRR-like protein kinase family (pthr27000), to the FLS2 sequences characterized in diverse plant species (NbFLS2, OsFLS2, CsFLS2-1 and CsFLS2-2, and VvFLS2) and to the sequences identified by Panther as AtFLS2 orthologs (ZmFLS2, HvFLS2, GmFLS2.1, and GmFLS2.2) (table S1). The multiple sequence alignment was performed using the ClustalW software with the following parameters: gap opening penalty: 10, gap extension penalty: 0.2, and 30% divergent cutoff. The evolutionary history was derived using the neighbor-joining (NJ) method with the bootstrap test (5000 replicates) in the MEGA X software. The optimal tree of the 31 amino acid sequences presented the sum of branch length = 11.96893034. The evolutionary distances were computed using the Poisson correction method and were in the units of the number of amino acid substitutions per site. All ambiguous positions were removed for each sequence pair (pairwise deletion option). There were a total of 1524 positions in the final dataset.

### RNA isolation and analysis by RT-qPCR of *V. faba* and *A. thaliana* tissue

The midrib and the rest of the leaf were separated before homogenization of both tissues in liquid nitrogen. RNA was isolated with TRIzol (Thermo Fisher Scientific, Dreieich, Germany) and chloroform according to the manufacturer’s protocol. The obtained RNA was further purified by precipitation in (1/10) of 3 M sodium acetate and 2 volumes of pure ethanol to remove salt and contaminants. Quality and quantity of RNA were analyzed photospectrometrically. One microgram of RNA was used for cDNA synthesis with RevertAid transcriptase (Thermo Fisher Scientific, Dreieich, Germany). Fifty nanograms of synthesized cDNA was used as a template for RT-qPCR in a CFX Connect Real-Time PCR Detection System (Bio-Rad, Munich, Germany) with EvaGreen (Biotium, Fremont, USA). The mRNA levels for each cDNA probe were normalized with respect to a plant housekeeping gene, *Actin2*, or *RPS18B* for *A. thaliana* and *PsGAPA* for *V. faba* [according to (*75*)]. All used RT-qPCR primers are listed in table S1. Fold induction values of target genes were calculated with the ∆∆CP equation and related to the mRNA level of target genes in the whole leaf sample, which was defined as 1.0. All assays were run with four biological replicates. *V. faba* gene-specific primers were designed using the NCBI (National Center for Biotechnology Information) primer design tool (www.ncbi.nlm.nih.gov/tools/primer-blast). Homologous sequences to the *AtFLS2* target gene were identified by blast against the v.faba_CSFL_reftransV2 dataset in Pulse Crop Database (https://pulsedb.org/). The identified contigs were v.faba_CSFL_reftrans V2_0007825 and v.faba_CSFL_reftrans V2_0001231.

### Cloning of a VfFLS2 fusion construct for agroinfiltration

Total RNA was isolated from *V. faba* leaf tissue using the innuPREP Plant RNA Kit (Analytik Jena, Jena, Germany) and reverse transcribed with PrimeScript RT Master Mix (Takara Bio, Kusatsu, Shiaga, Japan), and cDNA was used for the amplification of *VfFLS2* by PCR without translational stop codon using the forward primer AGAGTCGACATGTTATCATCTCTAAACCTTAACTTG and the reverse primer AGACCATGGATCTTGCCGTCTTAAGCTTCACG (restriction sites underlined). The PCR product was digested with the restriction endonucleases Sa lI and Nco I (New England Biolabs, Ipswich, Massachusetts, USA) and inserted into the corresponding restriction sites of the binary expression vector pLab12.10, containing the Cauliflower Mosaic Virus (CaMV) 35*S* promoter upstream of the inserted sequence and a C-terminal Venus tag for protein localization.

### Agroinfiltration of *N. benthamiana* plants

For subcellular localization, the *VfFLS2-Venus* fusion was coexpressed with a plasma membrane–located *CBL1-OFP* (*76*) or cytoplasmatic Cerulean in *N. benthamiana* epidermal cells. The *VfFLS2-Venus* construct and the plasmids carrying the subcellular markers were introduced into the *Agrobacterium tumefaciens* strain GV3101 pMP90 by electroporation. The transient expression was achieved by simultaneous infiltration of the respective plasmids and C58C1, carrying the pCH32 helper plasmid containing the RNA silencing suppressor protein p19 from tomato bushy stunt virus, into leaves of 4-week-old *N. benthamiana* plants. Infiltrated leaf discs were punched out after 3 days and analyzed by CLSM (TCS SP5, Leica, Germany). Fluorescence intensity at the given region of interest was detected using the following settings: Venus excitation wavelength 514 nm and emission wavelength 525 to 550 nm, OFP excitation wavelength 543 nm and emission wavelength 569 to 629 nm, and Cerulean excitation wavelength 458 nm and emission wavelength 469 to 503 nm.

### Bioassay with *A. thaliana* protoplasts

Leaf mesophyll protoplasts from 3- to 4-week-old *A. thaliana efr x fls2* mutant plants (*erf*-*fls2* elongation factor Tu receptor) were used for polyethylene glycol–calcium-mediated cotransformation of the pFRK1::luciferase reporter with the expression plasmid pLab12.10:p35S-VfFLS2-Venus. Protoplasts were resuspended in W5 solution containing 200 μM firefly luciferin (Synchem UG) and distributed in 96-well plates (20,000 protoplasts/90 μl). After overnight incubation at room temperature, protoplasts were treated with flg22 (10 times concentrated in W5 solution) or W5 as a mock control, and luciferase activity was recorded once per hour with a luminescence plate reader (Mithras LB 940, Berthold). Expression of VfFLS2 was verified by Western blot analysis (Fig. 5C) using anti-GFP antibodies (Torrey Pines) and secondary antibodies (Applied Biosystems) coupled to alkaline phosphatase with CDP-Star (Roche) as a substrate.

### Preparation of intact *V. faba* plants for microscopy and intracellular electrophysiology

#### Cutting a vascular observation window

For in vivo observation of intact sieve tubes of *V. faba* plants, cortical cell layers were locally removed by manual paradermal slicing from the abaxial side of the main vein of a mature leaf that was still attached to an intact plant (*36*). The intact phloem tissue in the created window was immersed in a bathing medium, whose composition was identical to the bathing medium described above for *A. thaliana*. The leaf was fixed to a microscope slide with double-sided adhesive tape and mounted onto the stage of a fluorescence microscope (AxioImager.M2, Carl Zeiss, Göttingen, Germany) equipped with an Axiocam 503 mono camera (Carl Zeiss) or a CLSM (TCS SP5, Leica, Germany). The intactness of the phloem tissue was checked microscopically using a water immersion objective (W N-ACHROPLAN 40x/0.75; Zeiss or HCX APO L40x0.80WU-V-l objective; Leica).

#### Observation of forisome reaction and sieve tube occlusion

For local treatments, the control bathing medium was replaced with a bathing medium containing flg22 (0.01, 0.1, 1, or 10 μM), glutamic acid (1 and 10 mM), GABA (5 and 50 mM), or sorbitol (1 M), and forisome reactions were monitored.

#### Intracellular electrophysiological measurements

Intracellular electrophysiological measurements were conducted as described by Furch *et al.* (*23*). Briefly, the exposed phloem tissue in the window was submersed in a bathing medium for 1 hour. Microelectrode tips with a diameter of 1 μm were impaled into SEs, phloem parenchyma cells, or subepidermal cells using an LN SM-1-micromanipulator (Luigs and Neumann, Ratingen, Germany) under continuous microscopic surveillance. After stabilization of the resting potential, 1 μM flg22 solution (diluted in a bathing medium) was applied to the observation window, whereas membrane potential profiles were recorded. The Ca^2+^ channel blocker La^3+^ was prepared as a stock solution (100 mM in a bathing medium) and diluted to a final concentration of 2 mM in a bathing medium. Incubation of phloem tissue with La^3+^ lasted between 2 and 3 hours before flg22 application was performed.

### Preparation of SE protoplasts from *V. faba* for osmotic shock experiments and ROS detection

Internodes were excised from 3- to 4-week-old *V. faba* plants and split radially into two halves (*35*). For coarse mechanical isolation of stem phloem strands, tangential tissue sheets with a thickness of ~300 μm were sliced from the fracture face of the split internode. After preincubation for 15 min in a protoplast standard medium (PSM) containing 600 mM mannitol, 1 mM dl-dithiothreitol (DTT), and 25 mM MES/NaOH (pH 5.7), the tissue slices were transferred to the enzyme mixture (400 mM mannitol, 100 mM KCl, 5 mM MgSO_4_, 1 mM DTT, 0.2% polyvinyl pyrrolidone, 0.5% bovine serum albumin, 0.55% Cellulase RS, 0.035% Pectolyase Y-23, and 25 mM MES/NaOH (pH 5.7)]. After incubation for 10 hours at 28°C, disintegrating phloem strands were filtered through an 80-μm nylon mesh, and the perfused protoplasts were washed twice with PSM. To check whether the forisome is responsive to flg22, the medium was replaced by a solution containing 1 mM CaCl_2_, 600 mM mannitol, or 1.25 μM flg22. A hypoosmotic solution containing 1 mM Ca^2+^ and 60 mM mannitol was applied for an intactness test of the protoplast. The effects were examined by a light microscope (AxioImager.M2, Carl Zeiss). To visualize flg22-induced accumulation of ROS in SE and subepidermal cell protoplasts, H_2_DCFDA (Invitrogen Molecular Probes, Eugene, OR, USA) was mixed with PSM to a final concentration of 10 μM. After incubation of 30 min, protoplasts were washed for 5 min with PSM. Observation of ROS-dependent H_2_DCFDA fluorescence was performed using a CLSM (SP2, Leica Microsystems, Heidelberg, Germany) at the 488-nm wavelength line after replacement of PSM by 1 μM flg22 dissolved in PSM.

### Extraction and quantification of phytohormones by LC-MS/MS

Leaves of 5- to 6-week-old *Arabidopsis* plants were counted (*31*), and leaf number 8 was treated (local spray) with 100 μl of 1 μM flg22 solution or water without treating or touching any other leaf. After incubation for the indicated time, leaf numbers 8 and 13 were harvested into liquid nitrogen and stored at −80°C until further use. The extraction procedure and phytohormone determination was performed according to Müller *et al.* (*77*). The frozen plant material was homogenized in a Geno/Grinder 2010 (Spex Sample Prep, Stanmore, UK) equipped with aluminum racks. Racks were cooled in liquid nitrogen before use to prevent thawing of the plant material throughout the homogenization process. Leaf tissue was extracted and homogenized in 1.5 ml of methanol containing internal standards: 60 ng of D4-SA (Santa Cruz Biotechnology, USA), 60 ng of D6-JA, and 12 ng of D6-JA-Ile (HPC Standards, Germany). Phytohormone analysis was performed by liquid chromatography–tandem mass spectrometry (LC-MS/MS) on an Agilent 1260 series HPLC system (Agilent Technologies, Waldbronn, Germany) coupled to a tandem mass spectrometer QTRAP 6500 (SCIEX, Darmstadt, Germany).

### Generation of *AtSEOR* double knockout *A. thaliana* mutants (*Atseor1/2*)

For the *AtSEOR* genes the nomenclature has changed over time. An earlier work refers to *AtSEOa* and *AtSEOb* (*63*), whereas in later publications, they are called *AtSEOR1* (*AtSEOb*) and *AtSEOR2* (*AtSEOa*) (*59*). These names are also used in this work.

Because of the clustered localization of the *AtSEOR* genes on chromosome 3 in the *A. thaliana* genome, it is almost impossible to generate a double knockout via crossing. Instead, a CRISPR-Cas9–mediated knockout was generated. The binary vector pDE-SauCas9 containing the Cas9 sequence from *Staphylococcus aureus* was kindly provided by H. Puchta (Karlsruhe Institute of Technology, Karlsruhe, Germany). For the construct, two specific protospacers (psp) that target a site in exon 1 of AtSEOR1 (CCAGCACGCTCAGCAACTG) and AtSEOR2 (TTGAGAGGGTCGGCCGTTGG), respectively, were derived. For generation of the protospacers the target online predictor CCTop (http://crispr.cos.uni-heidelberg.de) was used. The protospacers were fused to the single guide RNA (sgRNA) under the control of the Arabidopsis U6 small nuclear RNA (snRNA) promoters AtU6-26 and U6-29.

The sequence, including the *AtSEOR1* protospacer, the sgRNA, and the *Arabidopsis* U6-26 terminator followed by the U6-29 promoter and the *AtSEOR2* protospacer flanked by Bbs I restriction sites, was synthesized (Thermo Fisher Scientific), digested with Bbs I, and cloned into the Bbs I–linearized entry vector, pEn-Chimera (*78*). The cassette was then transferred into the destination vector pDE-SauCas9 (*78*) using Gateway LR Clonase II Enzyme Mix (Thermo Fisher Scientific). The destination vector, containing the construct of PAtU6-26–pspAtSEOR1–sgRNA–termAtU6-26–PAtU6-29–pspAtSEOR2–sgRNA, was transformed into the *A. tumefaciens* strain EHA105 by electroporation and used for the transformation of *A. thaliana* Col-0 plants by floral dip.

After selection with kanamycin, resistant seedlings were planted in soil and screened for Cas9 insertion and expression. Total RNA was isolated using the innuPREP Plant RNA kit (Analytik Jena, Jena, Germany). Residual genomic DNA was removed using the TURBO DNA-free kit (Thermo Fisher Scientific, Waltham, MA, USA), and the RNA was transcribed into cDNA using PrimeScript RT Master Mix (Perfect Real Time, Takara Bio Europe, Saint-Germain-en-Laye, France). Transgenic plants were identified by PCR targeting the Cas9 (SauCas9 fw AACAGGCAGACCAACGAGAG/SauCas9 rev CCCTTAGCGAGGTTGAGGA) with the cDNA as a template (MangoTaq DNA polymerase, Bioline).

Successfully transformed plants were then screened for genome editing. Genomic DNA was isolated and used as a template for PCR (Phusion DNA polymerase, Thermo Fisher Scientific) to amplify the DNA region corresponding to the protospacers. The amplicons resulting from the primer combinations PAtSEOR2 fw ATCAGGATAACTCGTTGTACCATC/AtSEOR2 rev ACAGAGAACGTCGTCGTGTTG and PAtSEOR1 fw AAACTCGCAACATTTCAGTGAACC/AtSEOR1 rev GGAGACACTATCAAGAACGCTCA were purified using the NucleoSpin Gel and PCR Clean-up Kit (Macherey-Nagel, Düren, Germany) and sequenced.

#### *Crossing of *Atseor1* CRISPR knockout with *Atseor2

Sequencing revealed only CRISPR events in the targeted *AtSEOR1* sequence, resulting in an *AtSEOR1* single knockout but not the desired double knockout. Following the premise that the line contains a functional CRISPR-Cas9 construct targeting *AtSEOR1*, it can be crossed with the established *Atseor2* single knockout line (*63*). Because of the clustered location of the two genes on the third chromosome, the crossing will not result in a double knockout line but in a line carrying the *Atseor2* knockout and a functional CRISPR-Cas9 construct, which can generate a knockout mutation in the *AtSEOR1* gene on the same chromosome where the *AtSEOR2* knockout is located. Homozygous plants of each line were used for the crossing. Self-pollination of the AtSEOR1 CRISPR line was prevented by emasculating the immature flowers. All flower organs except for the pistil were removed, and the flowers from the AtSEOR2 single knockout line were used for fertilization.

The seeds of the crossed plants were harvested and selected for kanamycin resistance. All plants were screened for Cas9 expression as described above. To increase the enzymatic activity of the Cas9, the plants were subjected to a repeated heat treatment. Briefly, 2-week-old seedlings were incubated at 37°C for 30 hours followed by a regeneration period of 42 hours at 22°C. The treatment was repeated four times, after which the plants were kept for seed production. The seeds were sown, and the T1 generation was screened for loss-of-function mutations in AtSEOR1.

Crucially, the mutation had to be located on the chromosome carrying the T-DNA insertion responsible for the AtSEOR2 knockout. Specific primers binding within the T-DNA insertion and downstream of the potential CRISPR event were used to ensure the amplification from the target chromosome (k3-T-DNA4335 fw ACCCCAGTACATTAAAAACGTCCG/AtSEOR1 rev GGAGACACTATCAAGAACGCTCA). The plants were screened by direct sequencing of the purified amplicons. As a template for the PCR, genomic DNA was extracted by magnetic beats in a 96-well plate format. The Chemagic DNA Plant kit (PerkinElmer, Waltham, MA, USA) was used in combination with a magnetic stand and rotary shaker for beat resuspension. This analysis identified a line carrying a 1–base pair insertion located on the same chromosome as the T-DNA insertion responsible for the AtSEOR2 knockout, resulting in a premature termination of AtSEOR1 synthesis after 30 amino acids. The second generation of the double knockout line was screened for homozygosity by PCR and sequencing of the purified amplicons.

### Pathogenicity assays with *A. thaliana*

*P. syringae* pv. tomato DC3000 were cultured as described by Richter *et al.* (*79*). The Pseudomonas solution was adjusted to an OD_600_ (optical density at 600 nm) of 0.2, which corresponds to 10^8^ colony-forming units (CFU)/ml and diluted 1:1000 to a concentration of 10^5^ CFU/ml.

The solution was infiltrated into *A. thaliana* leaves (*80*). For experiments 1 to 5, executed through a year, three leaves of each plant were infiltrated; 5 mM MgCl_2_ was used as a mock control. To achieve more homogeneous results, plants were grown for experiment 6 under short-day conditions, and the leaves were counted to identify the eight leaf of each plant, which was then used for infiltration. After infiltration, the plants were under the described conditions for 3 days before the infected leaves were harvested.

For quantification of the infection level, six leaf discs (5 mm) from each infected plant were punched out and washed in 70% ethanol followed by H_2_O for 1 min. Afterward, the samples were homogenized, and 1 ml of sterile 10 mM MgCl_2_ was added. A dilution series (dilution 1:50; 1:100; 1:500; 1:1000) was made from the leaf extract and plated in triplicate of 10 μl on nutrient yeast glycerol (NGY) plates [peptone from casein (5 g/liter), yeast extract (3 g/liter), glycerol (20 ml/liter), and agar (15 g/liter)]. After 2 to 3 days, the lowest countable dilution was counted and the values in CFU/cm^2^ of the infected leaves were calculated.

## Supplementary Materials

### This PDF file includes:

Figs. S1 to S11 Table S1 References
