# Supplementary material for: Transformation of flg22 perception into electrical signals decoded in vasculature leads to sieve tube blockage and pathogen resistance
Source: Sci Adv. 2025 Feb 26;11(9):eads6417. doi: 10.1126/sciadv.ads6417 (PMC11864193; doi:10.1126/sciadv.ads6417)
Supplement: Supplementary file 1 — Figs. S1 to S11 Table S1 References [file sciadv.ads6417_sm.pdf]

Science Advances
AAAS

Supplementary Materials for

Transformation of flg22 perception into electrical signals decoded in vasculature leads to sieve tube blockage and pathogen resistance

Alexandra C. U. Furch *et al.*

Corresponding author: Alexandra C. U. Furch, alexandra.furch@uni-jena.de

Sci. Adv. **11**, eads6417 (2025)
DOI: 10.1126/sciadv.ads6417

This PDF file includes:

Figs. S1 to S11
Table S1
References

Figure S1:

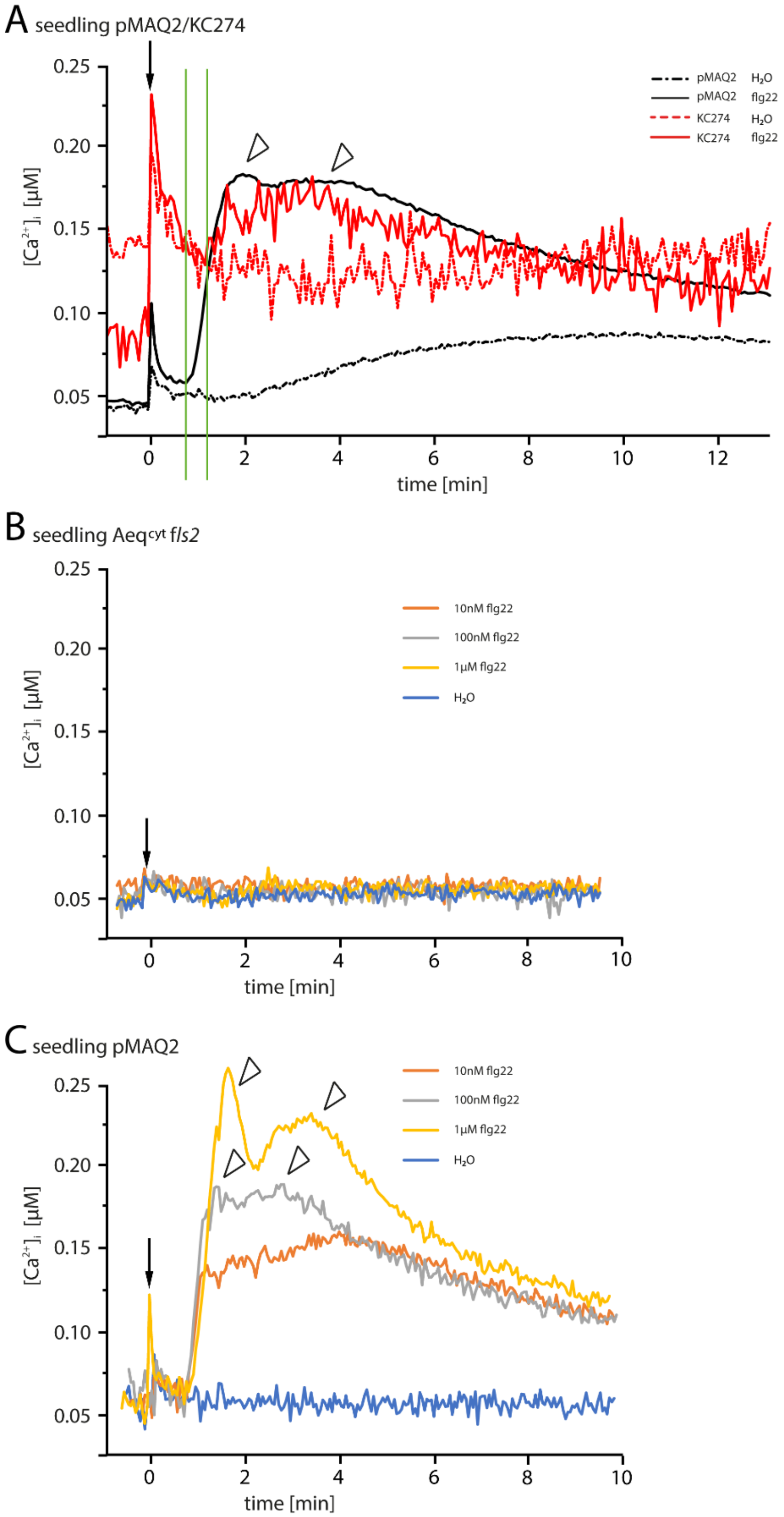
A seedling pMAQ2/KC274
pMAQ2 H₂O
pMAQ2 flg22
KC274 H₂O
KC274 flg22
[Ca²⁺]ᵢ [µM]
0.25
0.20
0.15
0.10
0.05
0
2
4
6
8
10
12
time [min]
B seedling Aeq^cyt fls2
10nM flg22
100nM flg22
1µM flg22
H₂O
[Ca²⁺]ᵢ [µM]
time [min]
C seedling pMAQ2
10nM flg22
100nM flg22
1µM flg22
H₂O
[Ca²⁺]ᵢ [µM]
time [min]

Fig. S1. Flg22-induced changes of $[Ca^{2+}]_{cyt}$ in vascular tissues and whole plant of *A. thaliana* seedlings. (A) A comparison of the time profile of $[Ca^{2+}]_{cyt}$ changes in the vascular system (vasculature GAL4 enhancer trap line KC274) and the collective cytosol of seedlings (pMAQ2) was made using a lowered (from 6s to 4s) measuring interval. Entire seedlings were treated with 1 µM flg22 or water, and the luminescence was recorded. After flg22 treatment, two successive $[Ca^{2+}]_{cyt}$ maxima (marked with arrowheads) were observed: the first peak occurred 1 min after flg22 application followed by a second, 3-4 min later (n=4). The time shifts of the Ca^{2+} response in either approach are marked with green vertical lines. (**B**) Time profile of $[Ca^{2+}]_{cyt}$ changes in the Aeq^{cyt}*fls2* line after different flg22 concentrations. (**C**) Comparison of the time profile of $[Ca^{2+}]_{cyt}$ changes in the pMAQ2 line after different flg22 concentrations. The two successive $[Ca^{2+}]_{cyt}$ maxima (marked with arrowheads) were found after 1 µM and 100 nM flg22 but not after 10 nM.

Figure S2.

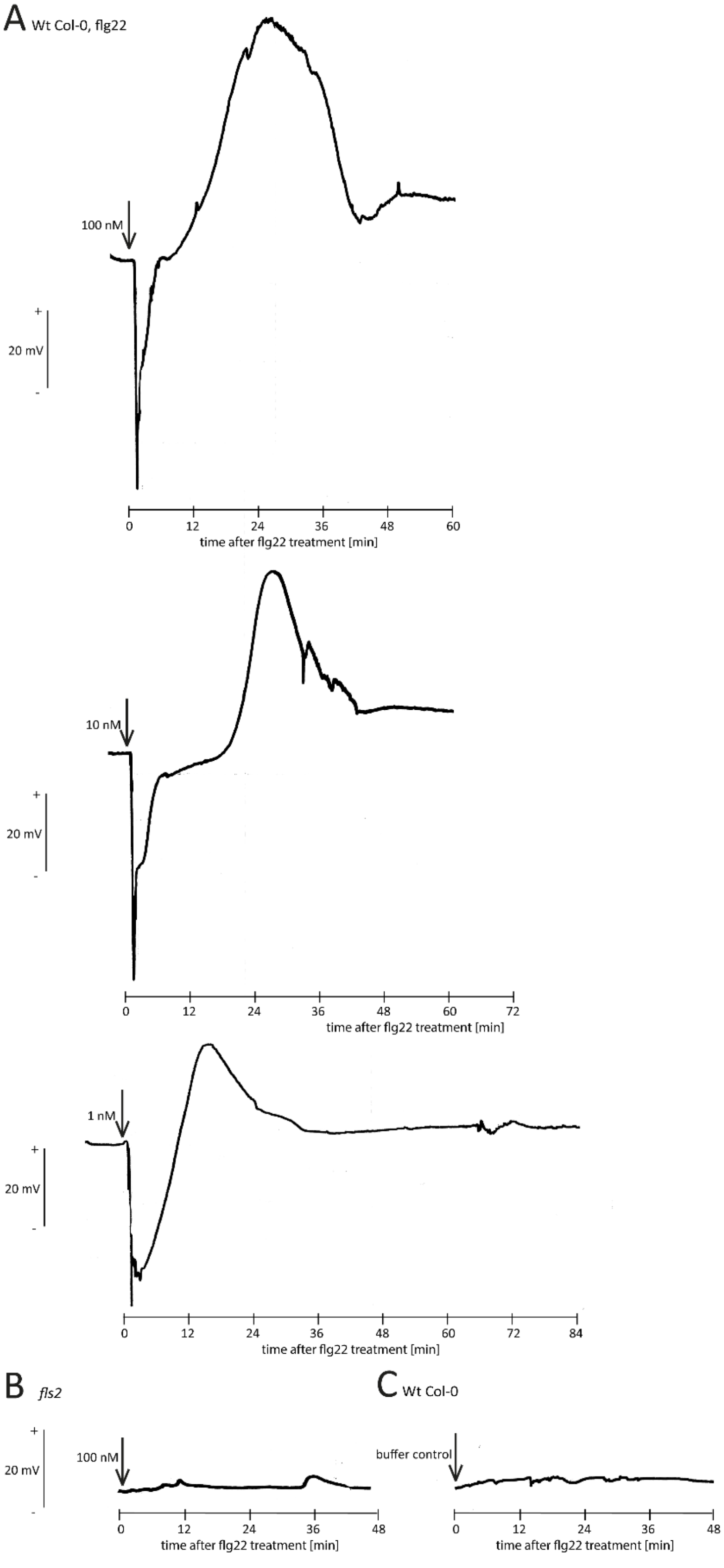

A Wt Col-0, flg22
100 nM
+
20 mV
-
0 12 24 36 48 60
time after flg22 treatment [min]
10 nM
+
20 mV
-
0 12 24 36 48 60 72
time after flg22 treatment [min]
1 nM
+
20 mV
-
0 12 24 36 48 60 72 84
time after flg22 treatment [min]
B fls2
+
20 mV
-
100 nM
0 12 24 36 48
time after flg22 treatment [min]
C Wt Col-0
buffer control
0 12 24 36 48
time after flg22 treatment [min]

Fig. S2. Extracellular voltage recordings in response to remote epidermal flg22 application onto the *A. thaliana* midrib. The flg22 solutions (1, 10 or 100 nM) or bathing medium (control) were carefully dropped onto the abaxial epidermis. (**A**) Flg22 induced voltage shifts in *A. thaliana* wild-type plants but not in (**B**) *fls2* mutant plants or (**C**) after a control treatment. Time points of flg22 application are marked with an arrow. Each measurement was repeated at least 4 times.

Figure S3:

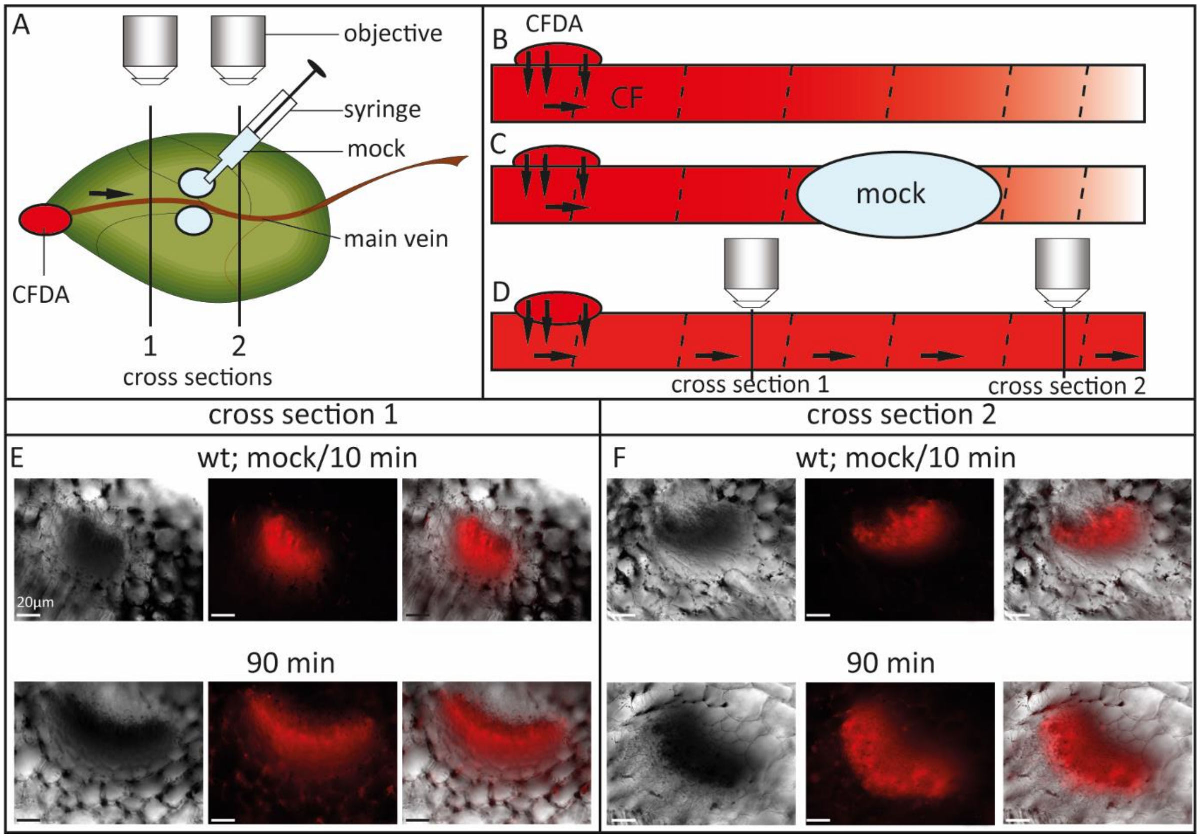
A
objective
syringe
mock
main vein
CFDA
1
2
cross sections
B
CFDA
CF
C
mock
D
cross section 1
cross section 2
cross section 1
cross section 2
E
wt; mock/10 min
20µm
90 min
F
wt; mock/10 min
90 min

Fig. S3. Fluorescence microscopic observations of phloem mass flow in *A. thaliana* WT after mock treatment as a control for flg22 application (see Fig. 4). (**A-D**) Schematic drawings of setup and SE reactions to application of bathing medium. (**A**) The non-fluorescent membrane-permeable ester 5(6)carboxyfluorescein diacetate (CFDA) was continuously applied to a cropped leaf tip and trapped by sieve elements (SEs). There, it was cleaved by esterases to form the polar (membrane-impermeable) fluorescent compound carboxyfluorescein (CF). Phloem transport of CF was observed by confocal laser scanning microscopy at cross-sections (vertical lines) upstream (1) and downstream (2) of the mock-solution infiltration site. The plants were treated with bathing medium (2 mM KCl, 1 mM $CaCl_2$, 1 mM $MgCl_2$, 50 mM mannitol and 2.5 mM MES/NaOH buffer, pH 5.7) without flg22. (**B**) CF was carried by mass flow through sieve tubes. (**C**) After 2 h, 100 µL of bathing medium was pressure-infiltrated via a 1 mL syringe, 0.5 cm at the right and left side of the midrib between the veins in an areole area. (**D**) At different time points after mock treatment (10 to 90 min), CF fluorescence in the phloem was examined in cross-sections up- (**E**) and downstream (**F**) of the infiltration site. (**E, F**) In all mock treated plants, CF fluorescence was always detected at both sides, up- and downstream the infiltration site (n=8). Transmission channel, fluorescence channel and merged images are presented from left to right (**E-F**).

Figure S4:

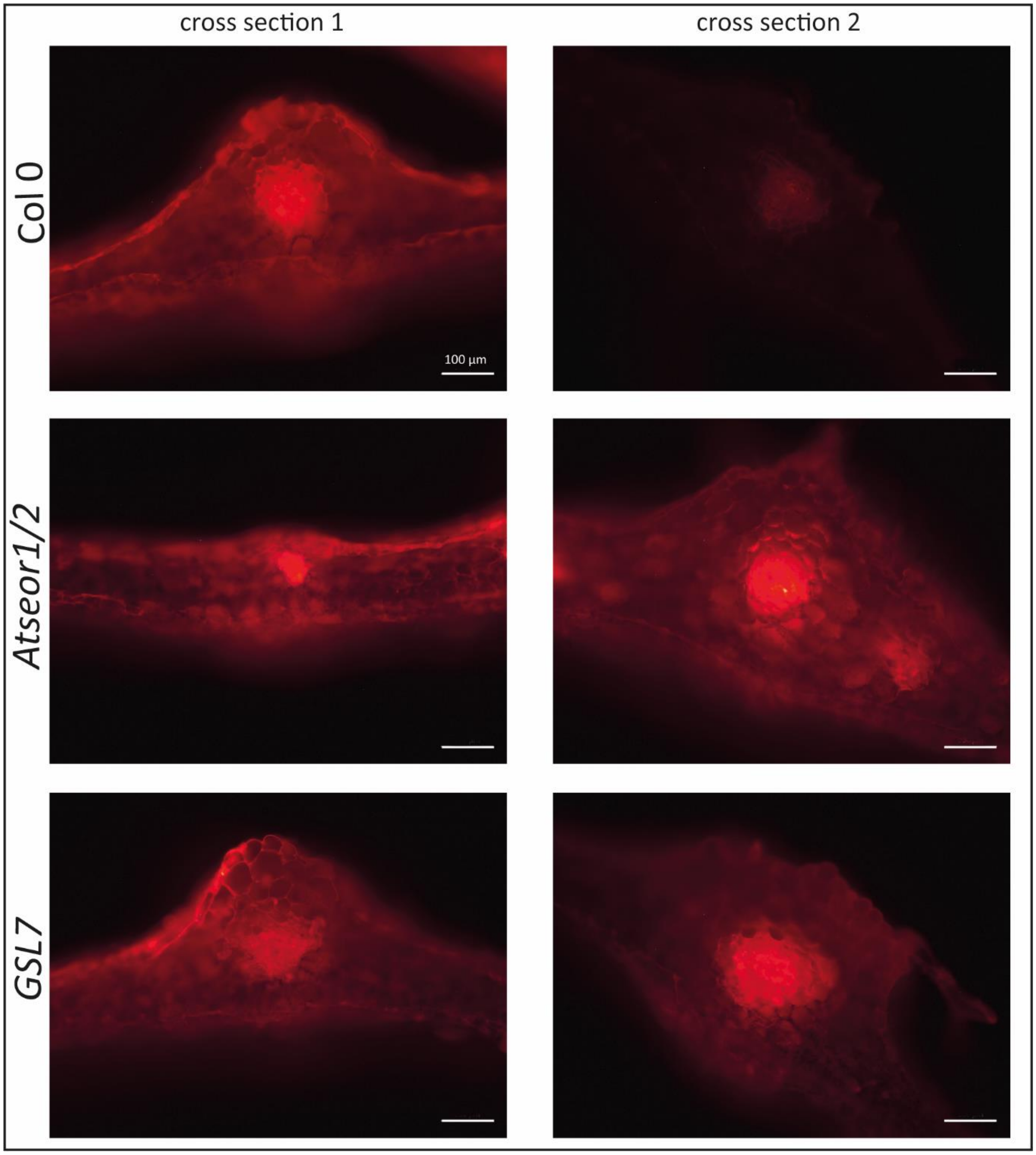

Fig. S4. Fluorescence microscopic observations of phloem mass flow in *A. thaliana* WT, *Atseor1/2 KO* and *Atgsl7 KO* mutants after 1 µM flg22 treatment. No CF fluorescence was observed downstream of the flg22 infiltration site of WT plants, indicating SEO (n=5), whereas CF fluorescence was always detected at both sides, up- and downstream the infiltration site of *Atseor1/2* and *Atgls7* mutants (n=5).

Figure S5:

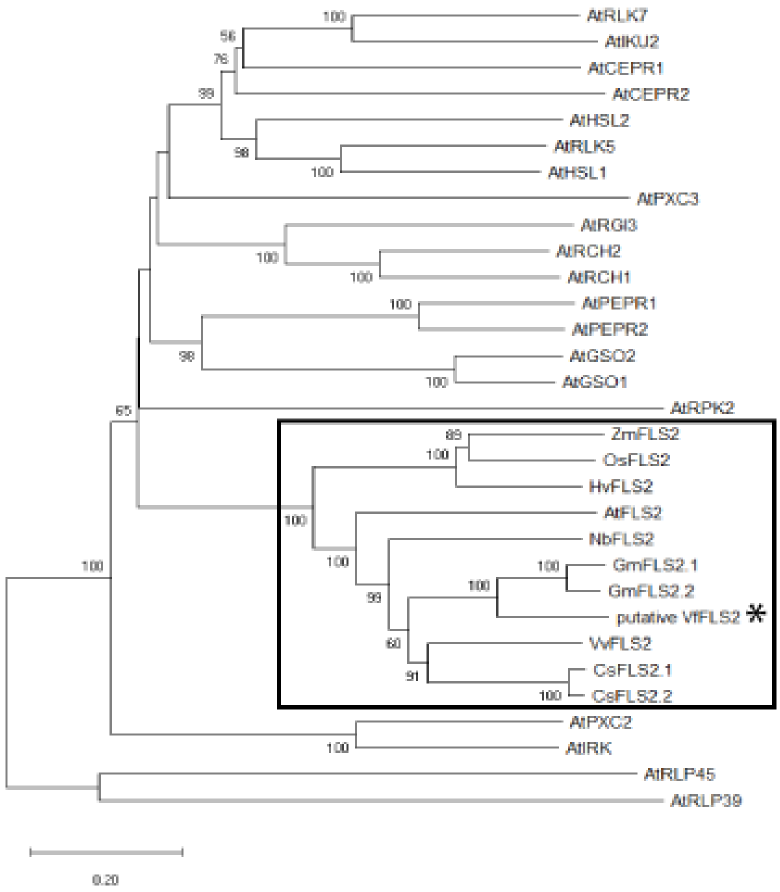

AtRLK7
AtIKU2
AtCEPR1
AtCEPR2
AtHSL2
AtRLK5
AtHSL1
AtPXC3
AtRGI3
AtRCH2
AtRCH1
AtPEPR1
AtPEPR2
AtGSO2
AtGSO1
AtRPK2
ZmFLS2
OsFLS2
HvFLS2
AtFLS2
NbFLS2
GmFLS2.1
GmFLS2.2
putative VfFLS2 *
VvFLS2
CsFLS2.1
CsFLS2.2
AtPXC2
AtIRK
AtRLP45
AtRLP39
0.20

Fig. S5. Phylogenetic analysis for FLS2 sequences of several plant species. The protein sequence of putative VfFLS2 was compared to the protein sequences of AtFLS2 and other 20 Arabidopsis members of the leucine-rich repeat receptor-like protein kinase family (pthr27000), to the FLS2 sequences characterized for different plant species (*Nb*FLS2, *Os*FLS2, *Cs*FLS2-1 and *Cs*FLS2-2, *Vv*FLS2) and to the sequences identified by Panther as *At*FLS2 orthologs (*Zm*FLS2, *Hv*FLS2, *Gm*FLS2.1 and *Gm*FLS2.2, *Cs*FLS2.1 and *Cs*FLS2.2). The percentage of replicate trees, in which the associated taxa are clustered in the bootstrap test, are shown next to the branches, only if bootstrap value was higher than 50. The tree is drawn to scale, with branch lengths in the same units as those of the evolutionary distances used to create the phylogenetic tree.

Figure S6:

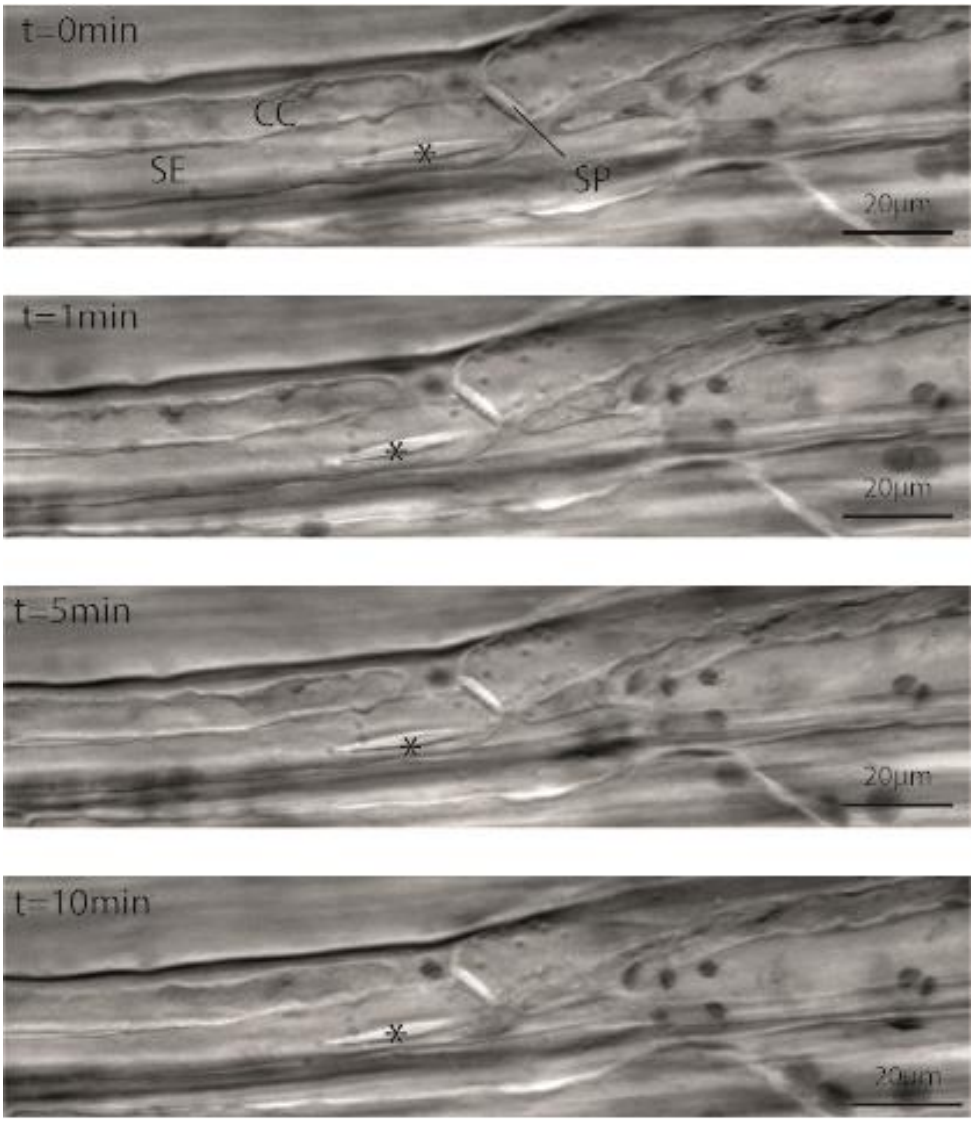

Fig. S6. Forisome reaction in response to a remote flg22 application in *V. faba*. At a distance of nearly 20 mm, 2 cortical windows were cut into the midrib of a *V. faba* leaf without damaging the vascular system. Both windows were bathed in a buffer solution in order to recover for 2 h. One window was used for the application of 1 µM flg22, and the second one was used for microscopic observation of forisome reaction. We never observed a forisome dispersion following a remote application of flg22. SE = sieve element; SP = sieve plate; * = forisome; t = time after application

Figure S7:

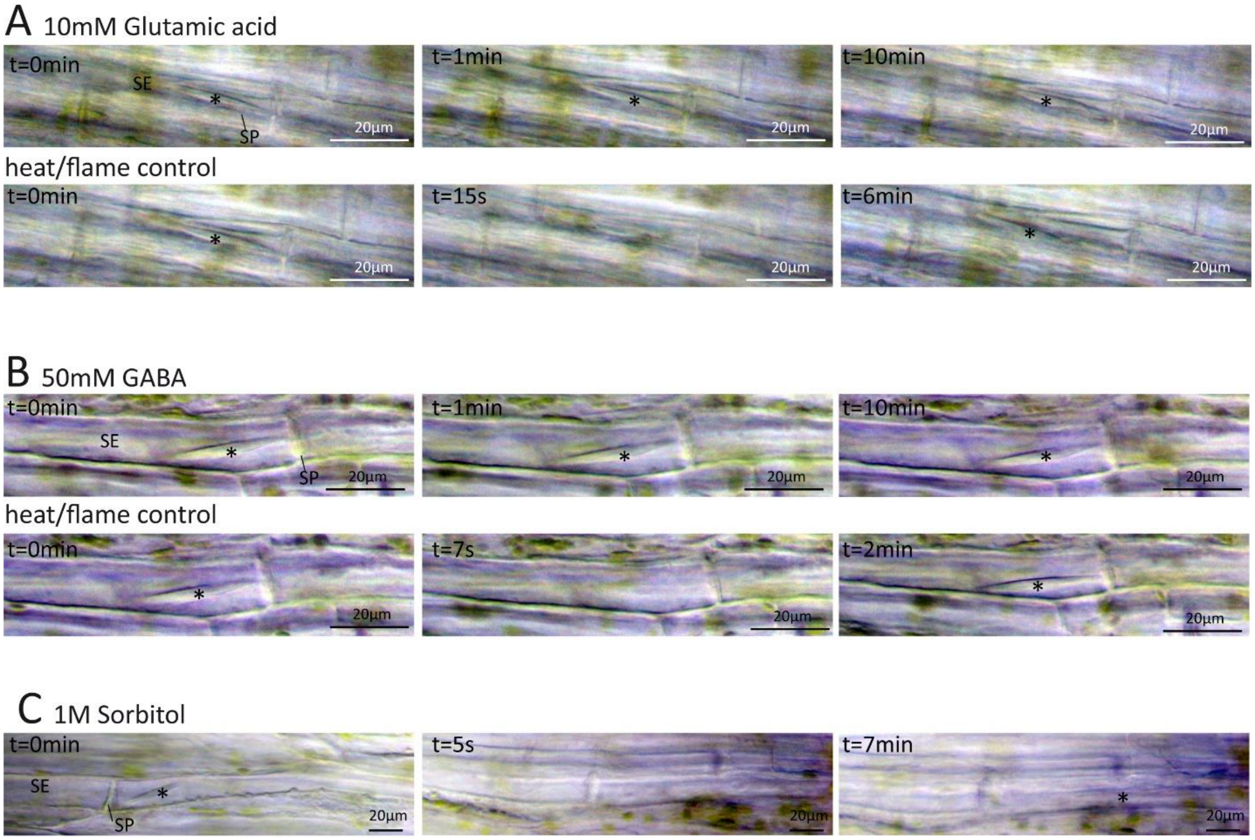

A 10mM Glutamic acid
t=0min
SE
*
SP
20µm
t=1min
t=10min
heat/flame control
t=15s
t=6min
B 50mM GABA
t=7s
t=2min
C 1M Sorbitol
t=5s
t=7min

Fig. S7. Forisome reaction in response to action potentials induced by local application of glutamic acid or GABA and variation potentials induced by sorbitol application in *V. faba*. The forisome reaction – dispersion and condensation – was microscopically monitored via observation windows made in the cortex (19). To this end, epidermis and cortical parenchyma cells of the midrib were removed with a fresh, sharp razor blade down to the vascular system without damaging the phloem. The tissue window was bathed into a buffer mock solution and recovered for 2h. Chemical stimuli – glutamic acid, γ-aminobutric acid (GABA), and sorbitol – were dissolved in the buffer solution and directly applied onto the tissue window. To check the intactness of the forisome, a heat stimulus was applied to the leaf apex at a distance of nearly 20 mm from the observation window. (**A, B**) Neither glutamic acid (1 or 10 mM; n = 4) nor GABA (5 or 50 mM; n = 4) induced a forisome dispersion (upper rows), while the remote heat stimulus always provided evidence for forisome viability (lower rows). (**C**) Forisome dispersion was triggered by a hyper-osmotic shock with 1 M sorbitol. The treatment seemingly damaged the plant tissue and a heat stimulus had no effect. SE = sieve element; SP = sieve plate; t = time after application; * = forisome

Figure S8:

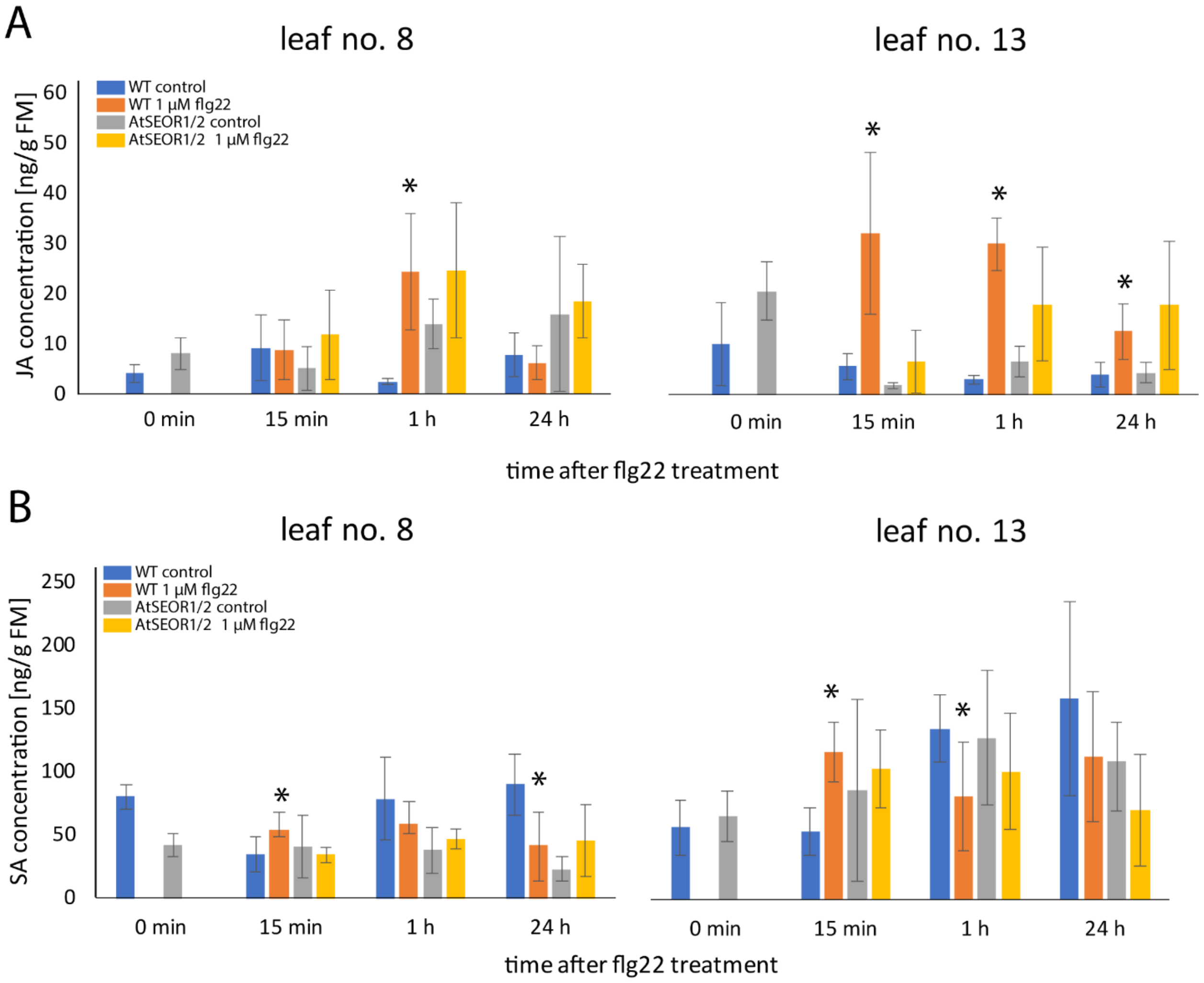
A
leaf no. 8
leaf no. 13
WT control
WT 1 µM flg22
AtSEOR1/2 control
AtSEOR1/2 1 µM flg22
JA concentration [ng/g FM]
0
10
20
30
40
50
60
0 min
15 min
1 h
24 h
*
time after flg22 treatment
B
leaf no. 8
leaf no. 13
WT control
WT 1 µM flg22
AtSEOR1/2 control
AtSEOR1/2 1 µM flg22
SA concentration [ng/g FM]
0
50
100
150
200
250
0 min
15 min
1 h
24 h
*
time after flg22 treatment

Fig. S8. Phytohormone determination in *A. thaliana* wild type (WT) and *Atseor1/2* double knockout plants upon flg22 treatment. Leaf 8 was treated with 1 µM flg22. Leaves 8 and 13 were harvested after the indicated time points and analyzed individually by LC-MS. (**A**) jasmonic acid (JA); (**B**) salicylic acid (SA). For all experiments, the bars represent the mean and standard error of the biological replicates (N=6). The asterisks indicate significant differences ($p<0.05$) between the control and treatment based on Student's t-Test.

Figure S9:

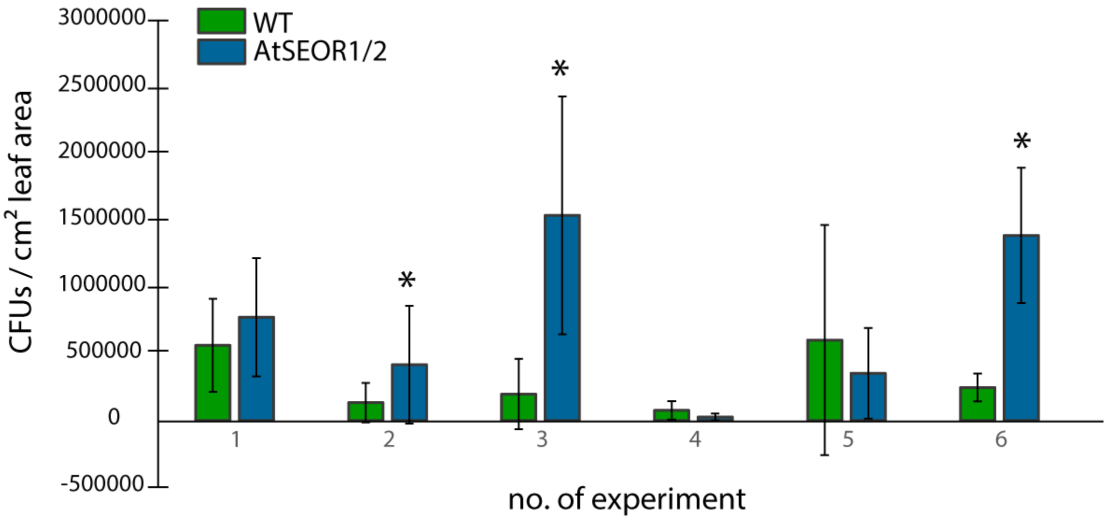
WT
AtSEOR1/2
CFUs / cm² leaf area
3000000
2500000
2000000
1500000
1000000
500000
0
-500000
1
2
3
4
5
6
*
*
*
no. of experiment

Fig. S9. Pathogen infection-related assays in *A. thaliana* wildtype (Col-0) and ΔAtSEOR1/2 mutant plants. *Pseudomonas syringae* infection level in *A. thaliana* leaves three days after infection. Leaves of WT (Col-0) (green) and the *ΔAtseor1/2* mutant line (blue) were infiltrated with *Pseudomonas syringae* pv tomato DC3000. Three days after the infiltration bacteria were isolated and grown on agar plates in different dilution Infection level (cfu/cm²) was determined by colony counting. The figure shows six experimental approaches (1-6). Sample sizes: 1 N=4; 2 $N=9_{WT}/8_{\Delta AtSEOR1/2}$; 3 N=6; 4 N=4; 5 N=12; 6 N=5. For all experiments, the bars represent the mean and standard error of the biological replicates. The asterisks indicate statistical significant($p<0.05$) between the WT and *ΔAtSEOR1/2* mutant line based on Student's t-Test.

Figure S10:

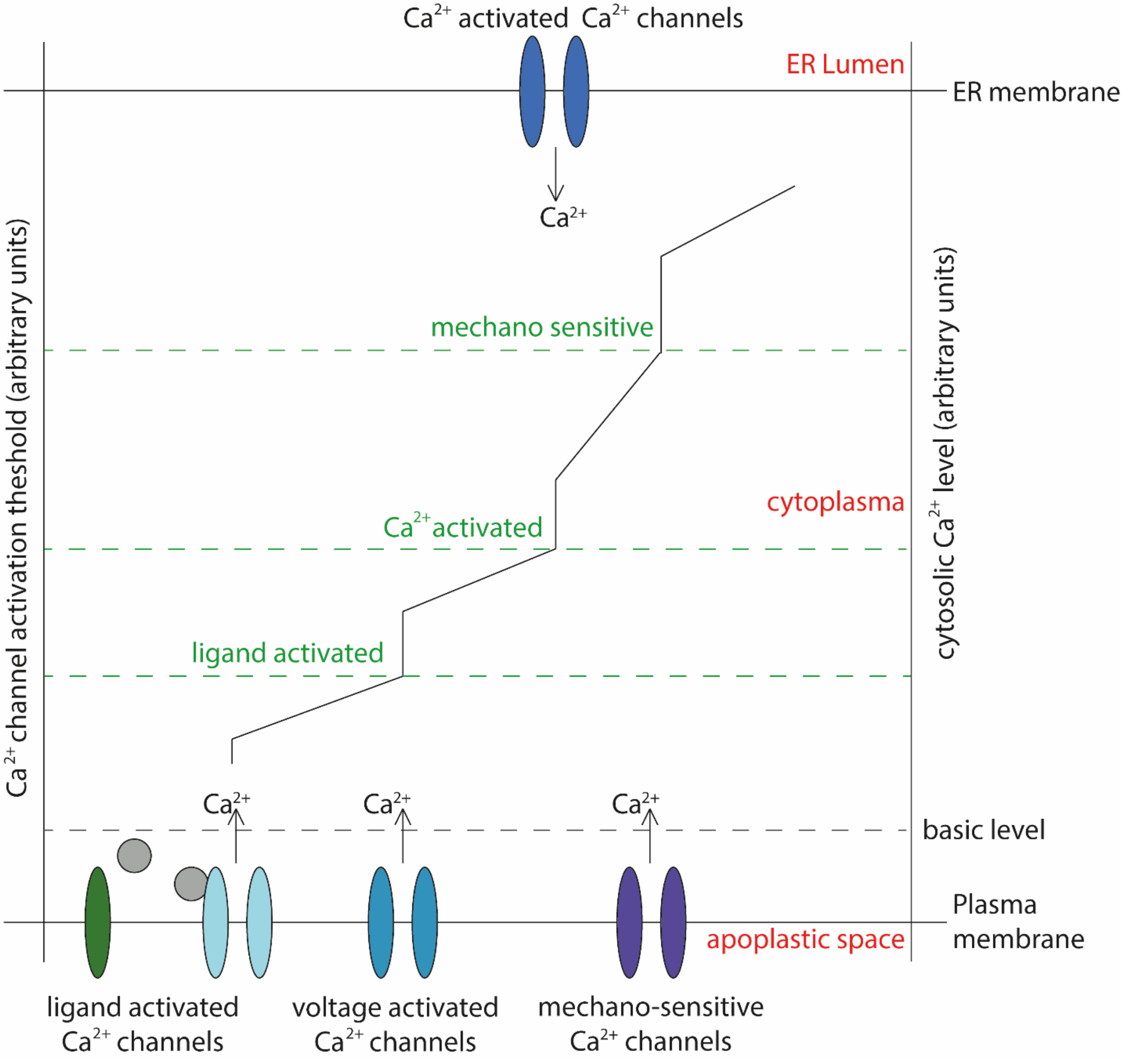

Ca2+ activated Ca2+ channels
ER Lumen
ER membrane
Ca2+
mechano sensitive
Ca2+ activated
cytoplasma
ligand activated
Ca2+ channel activation theshold (arbitrary units)
cytosolic Ca2+ level (arbitrary units)
Ca2+
Ca2+
Ca2+
basic level
Plasma membrane
apoplastic space
ligand activated Ca2+ channels
voltage activated Ca2+ channels
mechano-sensitive Ca2+ channels

Fig. S10. Hypothetical models of events in response to flg22 recognition by above-ground plant parts. Speculative model of a cascadic Ca^{2+} influx initiated by FLS2-mediated flg22 sensing in epidermal cells. The essence of the model is that successive gating of diverse ion channels with increasing gating threshold concentrations and Ca^{2+} transport capacities stepwise enhances cytosolic free Ca^{2+} concentration. Binding flg22 to FLS2 activates gating of ligand-activated Ca^{2+}-permeable channels located in the plasma membrane (lower abscissa). The resultant depolarisation activates voltage-activated Ca^{2+} permeable channels (lower abscissa) so that the cytosolic Ca^{2+} concentration is further enhanced to such an extent that Ca^{2+}-dependent Ca^{2+} permeable channels residing in the endomembrane system (upper abscissa) are gated. In turn, the extra Ca^{2+} elevation triggers such a massive release of ions that mechanosensitive Ca^{2+}-permeable channels in the plasma membrane (lower abscissa) are activated leading to a collapse of cell turgor. Involvement of voltage-activated channels initiates the propagation of an AP, while the engagement of mechanosensitive channels initiates a VPs. Ca^{2+} thresholds (stippled lines) are indicated on the left ordinate, Ca^{2+} influx (right ordinate) is given in arbitrary units.

Figure S11:

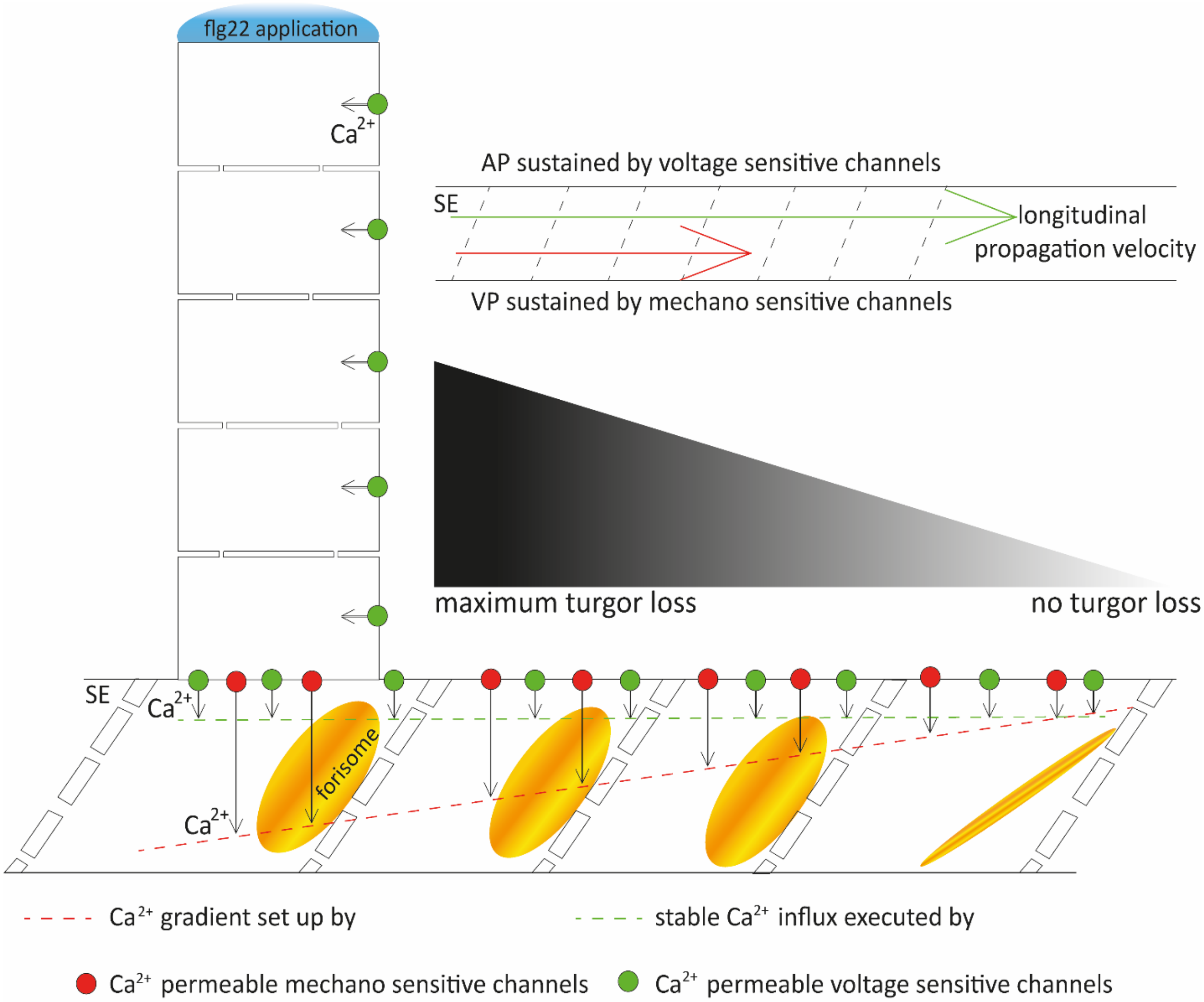

flg22 application
Ca2+
AP sustained by voltage sensitive channels
SE
longitudinal propagation velocity
VP sustained by mechano sensitive channels
maximum turgor loss
no turgor loss
SE
Ca2+
forisome
Ca2+
Ca2+ gradient set up by
stable Ca2+ influx executed by
Ca2+ permeable mechano sensitive channels
Ca2+ permeable voltage sensitive channels

Fig. S11. Origin, propagation modes and impact of successive electric potential waves. Model of two successive Ca^{2+} waves triggered by application of flg22 and transformed into an AP and VP, which appear near the site of infection as one merged EPW, but diverge further along the pathway. Divergence is due to the higher propagation speed of the AP. The AP is a self-amplifying EPW, enabled by voltage-activated Ca^{2+} channels located in the plasma membrane of SE/CCs, accomplishes a steady, but low Ca^{2+} influx below the occlusion threshold, but propagates more rapidly and over far longer distances than the VP. It impacts on the Ca^{2+} signatures of cells along the pathway and serves in this manner and act as defence-triggering alarm or priming signals to distant plant parts (43) that modulate phytohormone production. The VP is an electrical signal through SEs that originates from the mechanosensitive Ca^{2+} channels in SE-flanking cells. This is due to a wave of turgor loss, decreasing with the distance from the site of perception. As a consequence, Ca^{2+} influx into SEs and associate VPs fade with the distance travelled, and forisomes will disperse along the sieve-tubes until Ca^{2+} influx falls below the threshold for Ca^{2+}-induced forisome dispersion. This explains the distance-limited VP effect on forisome dispersion (Fig. 8). Only close to the site of flg22 application, the quantitative Ca^{2} influx exceeds the threshold needed for sieve plate occlusion, because Ca^{2+} influx via mechanosensitive Ca^{2+} channels decrease with the distance from the site of stimulation until final extinction.

Table S1.
Primers used for qRT-PCR.

Target	Sequence	Reference
AtActin2	**Fwd** 5´- GGAATCCACGAGACAACCTA -3´<br>**Rev** 5´- ATCTTCATGCTGCTTGGTGC -3´	70
AtFLS2	**Fwd** 5´- ACTCTCCTCCAGGGGCTAAGGAT -3′<br>**Rev** 5´- AGCTAACAGCTCTCCAGGGATGG -3′	81
AtRPS18B	**Fwd** 5´- GTCTCCAATGCCCTTGACAT -3′<br>**Rev** 5´- TCT TTCCTCTGCGACCAGTT -3′	82
AtSEOR1	**Fwd** 5´- TCCTAAGCCATCACTCGTCTTCA -3′<br>**Rev** 5´- CCGTATTTCACGGCCAAAGCA -3′	83
AtSEOR2	**Fwd** 5´- GGCCTTGGTTCATCCCAAACC -3′<br>**Rev** 5´- TGGAACCCACACAACCTCGTA -3′	65
PsGAPA	**Fwd** 5´- GATGGCATCTCAGTTGATGGAAAG -3´<br>**Rev** 5´- CTGTCCACAAACACTCCAGTTCCT -3´	75
VfFLS2	**Fwd** 5´- CGTTGCACACTTCAAAGGCA -3´<br>**Rev** 5´- CGCTTGTGCCATTTCCAACA -3´	this study

REFERENCES AND NOTES

1. T. Boller, G. Felix, A renaissance of elicitors: Perception of microbe-associated molecular pattern and danger signals by pattern-recognition receptors. *Annu. Rev. Plant Biol.* **60**, 379–406 (2009).

2. S. Ranf, L. Eschen-Lippold, P. Pecher, J. Lee, D. Scheel, Interplay between calcium signalling and early signalling elements during defence responses to microbe- or damage-associated molecular patterns. *Plant J.* **68**, 100–113 (2011).

3. J. Erickson, P. R. Weckwerth, T. Romeis, J. Lee, What's new in protein kinase/phosphatase signaling in the control of plant immunity? *Essays Biochem.* **66**, 621–634 (2022).

4. J. Zhang, J.-M. Zhou, Plant immunity triggered by microbial molecular signatures. *Mol. Plant* **3**, 783–793 (2010).

5. C. W. Melnyk, A. Molnar, D. C. Baulcombe, Intercellular and systemic movement of RNA silencing signals. *EMBO J.* **30**, 3553–3563 (2011).

6. F. Gaupels, A. C. U. Furch, T. Will, L. A. J. Mur, K.-H. Kogel, A. J. E. van Bel, Nitric oxide generation in *Vicia faba* phloem cells reveals them to be sensitive detectors as well as possible systemic transducers of stress signals. *New Phytol.* **178**, 634–646 (2008).

7. J. Durner, D. F. Klessig, Nitric oxide as a signal in plants. *Curr. Opin. Plant Biol.* **2**, 369–374 (1999).

8. H. W. Jung, T. J. Tschaplinski, L. Wang, J. Glazebrook, J. T. Greenberg, Priming in systemic plant immunity. *Science* **324**, 89–91 (2009).

9. K. Lorenc-Kukula, R. Chaturvedi, M. Roth, R. Welti, J. Shah, Biochemical and molecular-genetic characterization of SFD1's involvement in lipid metabolism and defense signaling. *Front. Plant Sci.* **2**, 26 (2012).

10. R. Chaturvedi, B. Venables, R. A. Petros, V. Nalam, M. Li, X. Wang, L. J. Takemoto, J. Shah, An abietane diterpenoid is a potent activator of systemic acquired resistance. *Plant J.* **71**, 161–172 (2012).

11. A. C. Vlot, D. A. Dempsey, D. F. Klessig, Salicylic acid, a multifaceted hormone to combat disease. *Annu. Rev. Phytopathol.* **47**, 177–206 (2009).

12. A. C. U. Furch, M. R. Zimmermann, K.-H. Kogel, M. Reichelt, A. Mithöfer, Direct and individual analysis of stress-related phytohormone dispersion in the vascular system of *Cucurbita maxima* after flagellin 22 treatment. *New Phytol.* **201**, 1176–1182 (2014).

13. L. Gomez-Gomez, G. Felix, T. Boller, A single locus determines sensitivity to bacterial flagellin in *Arabidopsis thaliana*. *Plant J.* **18**, 277–284 (1999).

14. M. Beck, I. Wyrsch, J. Strutt, R. Wimalasekera, A. Webb, T. Boller, S. Robatzek, Expression patterns of *FLAGELLIN SENSING 2* map to bacterial entry sites in plant shoots and roots. *J. Exp. Bot.* **65**, 6487–6498 (2014).

15. J. M. Smith, D. J. Salamango, M. E. Leslie, C. A. Collins, A. Heese, Sensitivity to flg22 is modulated by ligand-induced degradation and de novo synthesis of the endogenous flagellin-receptor FLAGELLIN-SENSING2. *Plant Physiol.* **164**, 440–454 (2014).

16. M. Giridhar, B. Meier, J. Imani, K.-H. Kogel, E. Peiter, U. C. Vothknecht, F. Chigri, Comparative analysis of stress-induced calcium signals in the crop species barley and the model plant *Arabidopsis thaliana*. *BMC Plant Biol.* **22**, 447 (2022).

17. D. Rissel, P. P. Heym, K. Thor, W. Brandt, L. A. Wessjohann, E. Peiter, No silver bullet-canonical Poly(ADP-Ribose) polymerases (PARPs) are no universal factors of abiotic and biotic stress resistance of *Arabidopsis thaliana*. *Front. Plant Sci.* **8**, 59 (2017).

18. L. Navarro, C. Zipfel, O. Rowland, I. Keller, S. Robatzek, T. Boller, J. D. G. Jones, The transcriptional innate immune response to flg22. Interplay and overlap with Avr gene-dependent defense responses and bacterial pathogenesis. *Plant Physiol.* **135**, 1113–1128 (2004).

19. A. C. U. Furch, A. J. E. van Bel, M. D. Fricker, H. H. Felle, M. Fuchs, J. B. Hafke, Sieve-element Ca^{2+} channels as relay stations between remote stimuli and sieve-tube occlusion in *Vicia faba*. *Plant Cell* **21**, 2118–2132 (2009).

20. M. R. Zimmermann, A. Mithöfer, "Electrical long-distance signaling in plants" in *Long-Distance Systemic Signalling and Communication in Plants*, F. Baluska, Ed. (Springer Verlag, 2013), pp. 291–308.

21. A. J. E. van Bel, A. C. U. Furch, T. Will, S. V. Buxa, R. Musetti, J. B. Hafke, Spread the news: Systemic dissemination and local impact of Ca^{2+} signals along the phloem pathway. *J. Exp. Bot.* **65**, 1761–1787 (2014).

22. M. R. Zimmermann, A. Mithöfer, T. Will, H. H. Felle, A. C. U. Furch, Herbivore-triggered electrophysiological reactions: Candidates for systemic signals in higher plants and the challenge of their identification. *Plant Physiol.* **170**, 2407–2419 (2016).

23. A. C. U. Furch, J. B. Hafke, A. Schulz, A. J. E. van Bel, Ca^{2+}-mediated remote control of reversible sieve-tube occlusion in *Vicia faba*. *J. Exp. Bot.* **58**, 2827–2838 (2007).

24. A. C. U. Furch, M. R. Zimmermann, T. Will, J. B. Hafke, A. J. E. van Bel, Remote-controlled stop of phloem mass flow by biphasic occlusion in *Cucurbita maxima*. *J. Exp. Bot.* **61**, 3697–3708 (2010).

25. S. Gilroy, N. Suzuki, G. Miller, W.-G. Choi, M. Toyota, A. R. Devireddy, R. Mittler, A tidal wave of signals: Calcium and ROS at the forefront of rapid systemic signaling. *Trends Plant Sci.* **19**, 623–630 (2014).

26. E. E. Farmer, Y.-Q. Gao, G. Lenzoni, J.-L. Wolfender, Q. Wu, Wound- and mechanostimulated electrical signals control hormone responses. *New Phytol.* **227**, 1037–1050 (2020).

27. E. Sukhova, V. Sukhov, Electrical signals, plant tolerance to actions of stressors, and programmed cell death: Is interaction possible? *Plants* **10**, 1704 (2021).

28. S. Robatzek, D. Chinchilla, T. Boller, Ligand-induced endocytosis of the pattern recognition receptor FLS2 in *Arabidopsis*. *Genes Develop.* **20**, 537–542 (2006).

29. K. Thor, S. Jiang, E. Michard, J. George, S. Scherzer, S. Huang, J. Dindas, P. Derbyshire, N. Leitão, T. A. DeFalco, P. Köster, K. Hunter, S. Kimura, J. Gronnier, L. Stransfeld, Y. Kadota,

C. A. Bücherl, M. Charpentier, M. Wrzaczek, D. MacLean, G. E. D. Oldroyd, F. L. H. Menke, M. R. G. Roelfsema, R. Hedrich, J. Feijó, C. Zipfel, The calcium-permeable channel OSCA1.3 regulates plant stomatal immunity. *Nature* **585**, 569–573 (2020).

30. D. H. Polisensky, J. Braam, Cold-shock regulation of the Arabidopsis TCH genes and the effects of modulating intracellular calcium levels. *Plant Physiol.* **111**, 1271–1279 (1996).

31. V. Kiep, J. Vadassery, J. Lattke, J.-P. Maaß, W. Boland, E. Peiter, A. Mithöfer, Systemic cytosolic Ca^{2+} elevation is activated upon wounding and herbivory in Arabidopsis. *New Phytol.* **207**, 996–1004 (2015).

32. M. C. Martí, M. A. Stancombe, A. A. R. Webb, Cell- and stimulus type-specific intracellular free Ca^{2+} signals in Arabidopsis. *Plant Physiol.* **163**, 625–634 (2013).

33. M. K. Paulmann, M. R. Zimmermann, L. Wegner, A. J. E. van Bel, G. Kunert, A. C. U. Furch, Species-specific and distance-dependent dispersive behaviour of forisomes in different legume species. *Int. J. Mol. Sci.* **22**, 492 (2021).

34. T. Will, A. J. E. van Bel, Physical and chemical interactions between aphids and plants. *J. Exp. Bot.* **57**, 729–737 (2006).

35. J. B. Hafke, A. C. U. Furch, M. U. Reitz, A. J. E. van Bel, Functional sieve element protoplasts. *Plant Physiol.* **145**, 703–711 (2007).

36. M. Knoblauch, A. J. E. van Bel, Sieve tubes in action. *Plant Cell* **10**, 35–50 (1998).

37. M. Knoblauch, G. A. Noll, T. Müller, D. Prüfer, I. Schneider-Hüther, D. Scharner, A. J. E. van Bel, W. S. Peters, ATP independent contractile proteins from plants. *Nat. Mater.* **2**, 600–603 (2003).

38. M. A. Torres, ROS in biotic interactions. *Physiol. Plant.* **138**, 414–429 (2010).

39. K. Thor, E. Peiter, Cytosolic calcium signals elicited by the pathogen-associated molecular pattern flg22 in stomatal guard cells are of an oscillatory nature. *New Phytol.* **204**, 873–881 (2014).

40. A. C. U. Furch, S. V. Buxa, A. J. E. van Bel, Similar intracellular location and stimulus reactivity, but differential mobility of tailless (*Vicia faba*) and tailed forisomes (*Phaseolus vulgaris*) in intact sieve tubes. *PLOS ONE* **10**, e0143920 (2015).

41. V. Vodeneev, E. Akinchits, V. Sukhov, Variation potential in higher plants: Mechanisms of generation and propagation. *Plant Signal. Behav.* **10**, e1057365 (2015).

42. H. H. Felle, M. R. Zimmermann, Systemic signaling in barley through action potentials. *Planta* **226**, 203–214 (2007).

43. G. Felix, J. D. Duran, S. Volko, T. Boller, Plants have a sensitive perception system for the most conserved domain of bacterial flagellin. *Plant J.* **18**, 265–276 (1999).

44. C. Zipfel, G. E. D. Oldroyd, Plant signaling in symbiosis and immunity. *Nature* **543**, 328–336 (2017).

45. M. Melotto, W. Underwood, S. Y. He, Role of stomata in plant innate immunity and foliar bacterial diseases. *Annu. Rev. Phytopathol.* **46**, 101–122 (2008).

46. J. He, N. Rössner, M. T. T. Hoang, S. Alejandro, E. Peiter, Transport, functions, and interaction of calcium and manganese in plant organellar compartments. *Plant Physiol.* **187**, 1940–1972 (2021).

47. D. Shkolnik, R. Nuriel, M. C. Bonza, A. Costa, H. Fromm, MIZ1 regulates ECA1 to generate a slow, long-distance phloem-transmitted Ca^{2+} signal essential for root water tracking in *Arabidopsis*. *Proc. Natl. Acad. Sci. U.S.A.* **115**, 8031–8036 (2018).

48. F. Resentini, M. Grenzi, D. Ancora, M. Cademartori, L. Luoni, M. Franco, A. Bassi, M. C. Bonza, A. Costa, Simultaneous imaging of ER and cytosolic Ca^{2+} dynamics reveals long-distance ER Ca^{2+} waves in plants. *Plant Physiol.* **187**, 603–617 (2021).

49. P. Ache, H. Bauer, H. Kollist, K. A. S. Al-Rasheid, S. Lautner, W. Hartung, R. Hedrich, Stomatal action directly feeds back on leaf turgor: New insights into the regulation of the plant water status from non-invasive pressure probe measurements. *Plant J.* **62**, 1072–1082 (2010).

50. W. Ye, Y. Murata, Microbe associated molecular pattern signaling in guard cells. *Front. Plant Sci.* **7**, 583 (2016).

51. W. S. Peters, K. H. Jensen, H. A. Stone, M. Knoblauch, Plasmodesmata and the problems with size: Interpreting the confusion. *J. Plant Physiol.* **257**, 153341 (2021).

52. A. C. Wille, W. J. Lucas, Ultrastructure and histological studies on guard cells. *Planta* **160**, 129–142 (1984).

53. A. Itaya, Y.-M. Woo, C. Masuta, Y. Bao, R. S. Nelson, B. Ding, Developmental regulation of intercellular protein trafficking through plasmodesmata in tobacco leaf epidermis. *Plant Physiol.* **118**, 373–385 (1998).

54. C. Faulkner, E. Petutschnig, Y. Benitez-Alfonso, M. Beck, S. Robatzek, V. Lipka, A. J. Maule, LYM2-dependent chitin perception limits molecular flux via plasmodesmata. *Proc. Natl. Acad. Sci. U.S.A.* **110**, 9166–9170 (2013).

55. N. De Storme, D. Geelen, Callose homeostasis of plasmodesmata; Molecular regulators and developmental relevance. *Front. Plant Sci.* **5**, 138 (2016).

56. E. Jeworutzki, M. R. G. Roelfsema, U. Anschütz, E. Krol, J. T. M. Elzenga, G. Felix, T. Boller, R. Hedrich, D. Becker, Early signalling through the Arabidopsis pattern recognition receptors FLS2 and EFR involves Ca^{2+}-associated opening of plasma membrane anion channels. *Plant J.* **62**, 367–378 (2010).

57. Q. Wu, Y. Li, M. Chen, X. Kong, Companion cell mediates wound-stimulated leaf-to-leaf electrical signalling. *Proc. Natl. Acad. Sci. U.S.A.* **121**, e2400639121 (2024).

58. E. E. Farmer, D. Gasperini, I. F. Acosta, The squeeze cell hypothesis for the activation of jasmonate synthesis in response to wounding. *New Phytol.* **204**, 282–288 (2014).

59. G. A. Noll, A. C. U. Furch, J. Rose, F. Visser, D. Prüfer, Guardians of the phloem—Forisomes and beyond. *New Phytol.* **236**, 1245–1260 (2022).

60. B. Xu, C. Cheval, A. Laohavisit, B. Hocking, D. Chiasson, T. S. G. Olsson, K. Shirasu, C. Faulkner, M. Gilliham, A calmodulin-like protein regulates plasmodesmal closure during bacterial immune responses. *New Phytol.* **215**, 77–84 (2017).

61. M. Grenzi, S. Buratti, A. S. Parmagnani, I. A. Aziz, I. Bernacka-Wojcik, F. Resentini, J. Šimura, F. G. Doccula, A. Alfieri, L. Luoni, K. Ljung, M. C. Bonza, E. Stavrinidou, A. Costa, Long-distance turgor pressure changes induce local activation of plant glutamate receptor-like channels. *Curr. Biol.* **33**, 1019–1035.e8 (2023).

62. D. R. Froelich, D. L. Mullendore, K. H. Jensen, T. J. Ross-Elliott, J. A. Anstead, G. A. Thompson, H. C. Pélissier, M. Knoblauch, Phloem ultrastructure and pressure flow: Sieve-element-occlusion-related agglomerations do not affect translocation. *Plant Cell* **23**, 4428–4445 (2011).

63. S. B. Jekat, A. M. Ernst, A. von Bohl, S. Zielonka, R. M. Twyman, G. A. Noll, D. Prüfer, P-proteins in *Arabidopsis* are heteromeric structures involved in rapid sieve tube sealing. *Front. Plant Sci.* **4**, 225 (2013).

64. X. Wang, L. Cheng, W. Peng, G. Xie, Z. Liu, F. An, Identification of sieve element occlusion gene (SEOs) family in rubber trees (*Hevea brasiliensis* Muell. Arg.) provides insights to the mechanism of Laticifer plugging. *Forests* **13**, 433 (2022).

65. L. Pagliari, S. Buoso, S. Santi, A. C. U. Furch, M. Martini, F. Degola, A. Loschi, A. J. E. van Bel, R. Musetti, Filamentous sieve element proteins are able to limit phloem mass flow, but not phytoplasma spread. *J. Exp. Bot.* **68**, 3673–3688 (2017).

66. Y.-Q. Gao, P. Jimenez-Sandoval, S. Tiwari, S. Stolz, J. Wang, G. Glauser, J. Santiago, E. E. Farmer, Ricca's factors as mobile proteinaceous effectors of electrical signalling. *Cell* **186**, 1337–1351.e20 (2023).

67. J. Shah, R. Chaturvedi, Z. Chowdhury, B. Venables, R. A. Petros, Signaling by small metabolites in systemic acquired resistance. *Plant Cell* **79**, 645–658 (2014).

68. A. J. K. Koo, X. Gao, A. D. Jones, G. A. Howe, A rapid wound signal activates the systemic synthesis of bioactive jasmonates in Arabidopsis. *Plant J.* **59**, 974–986 (2009).

69. M. Heyer, M. Reichelt, A. Mithöfer, A holistic approach to analyze systemic jasmonate accumulation in individual leaves of *Arabidopsis* rosettes upon wounding. *Front. Plant Sci.* **9**, 1569 (2018).

70. J. M. Johnson, J. Thürich, E. K. Petutschnig, L. Altschmied, D. Meichsner, I. Sherameti, J. Dindas, A. Mrozinska, C. Paetz, S. S. Scholz, A. C. U. Furch, V. Lipka, R. Hedrich, B. Schneider, A. Svato¡, R. Oelmüller, A poly(A) ribonuclease controls the cellotriose-based interaction between *Piriformospora indica* and its host Arabidsopsis. *Plant Physiol.* **176**, 2496–2514 (2018).

71. R. Ursache, T. Grube Andersen, P. Marhavý, N. Geldner, A protocol for combining fluorescent proteins with histological strains for diverse cell wall components. *Plant J.* **93**, 399–412 (2018).

72. N. Schnieder, A. Känel, M. Zimmermann, K. Kriebs, A. Witte, L. S. Wrobel, R. M. Twyman, D. Prüfer, A. C. U. Furch, G. A. Noll, So similar yet so different: The distinct contributions of extrafascicular and fascicular phloem to transport and exudation in cucumber plants. *J. Plant Physiol.* **271**, 153643 (2022).

73. H. H. Felle, S. Hanstein, The apoplastic pH of the substomatal cavity of *Vicia faba* leaves and its regulation responding to different stress factors. *J. Exp. Bot.* **53**, 73–82 (2002).

74. T. L. Bailey, M. Boden, F. A. Buske, M. Frith, C. E. Grant, L. Clementi, J. Ren, W. W. Li, W. S. Noble, MEME Suite: Tools for motif discovery and searching. *Nucleic Acids Res.* **37**, W202–W208 (2009).

75. N. Gutierrez, M. J. Giménez, C. Palomino, C. M. Avila, Assessment of candidate6reference genes for expression studies in *Vicia faba* L. by real-time quantitative PCR. *Mol. Breeding* **28**, 13–24 (2011).

76. O. Batistič, N. Sorek, S. Schültke, S. Yalovsky, J. Kudla, Dual fatty acyl modification determines the localization and plasma membrane targeting of CBL/CIPK Ca^{2+} signaling complexes in *Arabidopsis*. *Plant Cell* **20**, 1346–1362 (2008).

77. A. T. Müller, M. Reichelt, E. G. Cosio, N. Salinas, A. Nina, D. Wang, H. Moossen, H. Geilmann, J. Gershenzon, T. G. Köllner, A. Mithöfer, Combined –omics framework reveals how ant symbionts benefit the Neotropical ant-plant *Tococa quadrialata* at different levels. *iScience* **25**, 105261 (2022).

78. F. Fauser, S. Schiml, H. Puchta, Both CRISPR/Cas-based nucleases and nickases can be used efficiently for genome engineering in *Arabidopsis thaliana*. *Plant J.* **79**, 348–359 (2014).

79. C. Richter, M. E. Dirks, C. Schulze Gronover, D. Prüfer, B. M. Moerschbacher, Silencing and heterologous expression of *ppo-2* indicate a specific function of a single polyphenol oxidase isoform in resistance of dandelion (*Taraxacum officinale*) against *Pseudomonas syringae* pv. *tomato*. *Mol. Plant Microbe Interact.* **25**, 200–210 (2012).

80. F. Katagiri, R. Thilmony, S. Y. He, "The *Arabidopsis thaliana-Pseudomonas syringae* interaction" in *The Arabidopsis Book* (American Society of Plant Biologists, 2002).

81. F. Boutrot, C. Segonzac, K. N. Chang, H. Qiao, J. R. Ecker, C. Zipfel, J. P. Rathjen, Direct transcriptional control of the Arabidopsis immune receptor FLS2 by the ethylene-dependent transcription factors EIN3 and EIL1. *Proc. Natl. Acad. Sci. U.S.A.* **107**, 14502–14507 (2010).

82. S. S. Scholz, J. Vadassery, M. Heyer, M. Reichelt, K. W. Bender, W. A. Snedden, W. Boland, A. Mithöfer, Mutation of the *Arabidopsis* calmodulin-like protein CML37 deregulates the jasmonate pathway and enhances susceptibility to herbivory. *Mol. Plant* **7**, 1712–1726 (2014).

83. J. A. Anstead, D. R. Froelich, M. Knoblauch, G. A. Thompson, Arabidopsis p-protein filament formation requires both AtSEOR1 and AtSEOR2. *Plant Cell Physiol.* **53**, 1033–1042 (2012).
